# The USP10-HDAC6 axis confers cisplatin resistance in non-small cell lung cancer lacking wild-type p53

**DOI:** 10.1038/s41419-020-2519-8

**Published:** 2020-05-07

**Authors:** Chen Hu, Mu Zhang, Niko Moses, Cong-li Hu, Lisa Polin, Wei Chen, Hyejeong Jang, Joshua Heyza, Agnes Malysa, Joseph A. Caruso, Shengyan Xiang, Steve Patrick, Paul Stemmer, Zhenkun Lou, Wenlong Bai, Chuangui Wang, Gerold Bepler, Xiaohong Mary Zhang

**Affiliations:** 1 Department of Oncology, Karmanos Cancer Institute, Wayne State University, 4100 John R. St., Detroit, MI 48201 USA; 2 Institute of Translational Medicine, Shanghai General Hospital, Shanghai Jiao Tong University School of Medicine, 201620 Shanghai, China; 3 Cancer Biology Graduate Program, Karmanos Cancer Institute, 4100 John R. St., Detroit, MI 48201 USA; 4 Proteomics Facility Core, Institute of Environmental Health Sciences, Wayne State University, Scott Hall of Medical Sciences, 540 East Canfield, Room 2105, Detroit, MI 48201 USA; 5 Department of Pathology and Cell Biology, Morsani College of Medicine, University of South Florida, 12901 Bruce B. Downs Blvd., Tampa, FL 33612 USA; 6 Department of Oncology, Mayo Clinic, Rochester, MN 55905 USA; 7 Present Address: Institute for Quantitative Health Science and Engineering, Michigan State University, East Lansing, MI USA

**Keywords:** Enzyme mechanisms, Predictive markers

## Abstract

Ubiquitin-specific peptidase 10 (USP10) stabilizes both tumor suppressors and oncogenes in a context-dependent manner. However, the nature of USP10’s role in non-small cell lung cancer (NSCLC) remains unclear. By analyzing The Cancer Genome Atlas (TCGA) database, we have shown that high levels of USP10 are associated with poor overall survival in NSCLC with mutant p53, but not with wild-type p53. Consistently, genetic depletion or pharmacological inhibition of USP10 dramatically reduces the growth of lung cancer xenografts lacking wild-type p53 and sensitizes them to cisplatin. Mechanistically, USP10 interacts with, deubiquitinates, and stabilizes oncogenic protein histone deacetylase 6 (HDAC6). Furthermore, reintroducing either USP10 or HDAC6 into a USP10-knockdown NSCLC H1299 cell line with null-p53 renders cisplatin resistance. This result suggests the existence of a “USP10-HDAC6-cisplatin resistance” axis. Clinically, we have found a positive correlation between USP10 and HDAC6 expression in a cohort of NSCLC patient samples. Moreover, we have shown that high levels of USP10 mRNA correlate with poor overall survival in a cohort of advanced NSCLC patients who received platinum-based chemotherapy. Overall, our studies suggest that USP10 could be a potential biomarker for predicting patient response to platinum, and that targeting USP10 could sensitize lung cancer patients lacking wild-type p53 to platinum-based therapy.

## Introduction

Ubiquitin-specific peptidase 10 (USP10) belongs to the USP family^1^. The role of USP10 in cancer has remained elusive and complex, due to the diversity of its associated proteins and substrates^2–11^. For example, USP10 stabilizes a variety of tumor suppressors (i.e. PTEN, p14ARF, and p53)^5,12,13^, but is also reported to stabilize oncogenic FLT3, Slug/SNAI2, Raf1, Musashi-2, and is associated with enhanced G3BP2-mediated nuclear export of p53^14–18^. As for USP10’s tumor type-specific role, we suspect that USP10 behaves similarly to Notch and TGFβ. The former serves as an oncogene in leukemia and a tumor suppressor in head and neck tumors^19,20^; the latter servers as an oncogene in breast cancer and a tumor suppressor in colon cancer^21,22^. Likewise, USP10 plays an oncogenic role in breast cancer^23^, glioblastoma^24^, and prostate cancer^15^, and serves as a tumor suppressor role in renal cell^5^, gastric^25^, and pancreatic cancers^26^.

HDAC6 belongs to the class IIb HDAC family^27,28^. It is the most unique HDAC in that it contains two functional tandem deacetylase domains and a C-terminal ZnF-UBP domain^29^. HDAC6 deacetylates a wide array of proteins, including α-tubulin^30^, cortactin^31^, Hsp90^32^, and MSH2^33^. Dysregulation of HDAC6’s deacetylase activity and HDAC6 overexpression have both been associated with cancer and cisplatin resistance^34,35^. Moreover, HDAC6 protein is subject to ubiquitin-proteasome degradation pathway. Recently, Cullin 3^SPOP^ ubiquitin E3 ligase has been found to promote the poly-ubiquitination-mediated degradation of HDAC6^36^. However, the deuqbiquitinating enzyme responsible for stabilizing HDAC6 is completely unknown. Because of the oncogenic role of HDAC6, it is conceivable that the deubiquitinating enzymes stabilizing HDAC6 could contribute to cancer.

Here, we have identified USP10 as a deubiquitinating enzyme (DUB) for HDAC6. Overexpression of both USP10 and HDAC6 has been observed in lung and ovarian cancer. USP10 promotes lung cancer xenografts growth and confers cisplatin resistance in a mouse model. Moreover, our clinical data indicate that high levels of USP10 mRNA are associated with lower overall survival in advanced lung cancer patients who received platinum-based chemotherapy. Therefore, developing clinically relevant USP10 inhibitors could benefit lung cancer patients resistant to conventional platinum-based therapeutics.

## Results

### USP10 interacts with HDAC6

To investigate the role of USP10 in lung cancer, we employed a protein purification approach. Briefly, we established a Flag-HA-USP10 stable expressing H1299 cell line and purified USP10-associated proteins by chromatography-tandem mass spectrometry (LC-MS/MS). As shown in Table S1, we found 68 USP10 binding partners, which fall into eight functional groups (stress granules, RNA splicing factors, translational factors, protein stability, DNA repair and apoptosis, protein folding, cytoskeleton, and metabolism). One HDAC6 peptide (LVDAVLGAEIR) was identified from the above mass spectrometry analysis. Therefore, HDAC6 is a potential novel USP10-binding protein.

**Fig. 1:**
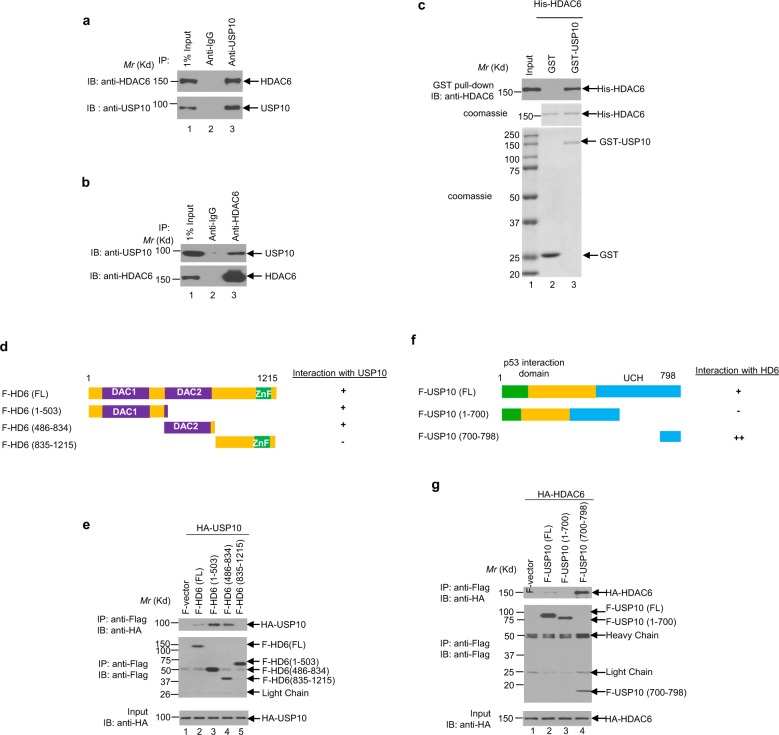
USP10 interacts with HDAC6. **a**, **b** Endogenous USP10 and HDAC6 interact with each other in H23 cells. **a** The anti-USP10 antibody or anti-IgG was used to immunoprecipitate USP10 in H23 cells followed by anti-HDAC6 Western blotting analysis (upper panel). The blot was stripped and reprobed with the anti-USP10 antibody (lower panel). **b** The reciprocal immunoprecipitation of **a** was performed. **c** USP10 physically binds to HDAC6. Bacterially-purified GST or GST-USP10 was incubated with His-HDAC6 isolated from *E.coli*, and the GST pull-down assays were performed followed by anti-HDAC6 Western blotting analysis (upper panel). His-HDAC6, GST, and GST-USP10 proteins were visualized via Coomassie blue staining (middle and lower panels). **d**, **e** Both the DAC1 domain and DAC2 domain of HDAC6 interact with USP10. **d** The schematic diagrams of HDAC6 full-length (FL) and deletion mutants. **e** The indicated vector and HDAC6 plasmids were co-transfected with HA-USP10 in 293T cells, followed by anti-Flag immunoprecipitation and subsequent anti-HA Western blotting analysis (upper panel). The blot was then stripped and reprobed with anti-Flag antibodies (middle panel). The input of HA-USP10 is shown in the lower panel. **f**, **g** The very C-terminus of USP10 binds to HDAC6. **f** The schematic diagrams of USP10 full-length (FL) and deletion mutants. **g** The indicated vector and USP10 plasmids were co-transfected with HA-HDAC6 in 293T cells, and anti-Flag immunoprecipitation was performed followed by anti-HA Western blotting analysis (upper panel). The blot was then reprobed with anti-Flag antibodies (middle panel). The input of HA-HDAC6 is shown in the lower panel.

To further confirm this interaction, we immunoprecipitated endogenous HDAC6 and USP10 in the H23 NSCLC cell line. As shown in Fig. 1a, the anti-USP10 antibody, but not the anti-lgG antibody, immunoprecipitated HDAC6. In the reciprocal experiment, the anti-HDAC6 antibody was able to immunoprecipitate USP10 (Fig. 1b). These results indeed verify that endogenous USP10 and HDAC6 interact with each other in lung cancer cells. To determine whether USP10 binds to HDAC6 directly or via other proteins, we generated and purified recombinant GST-USP10 and His-HDAC6 in *E. coli*. As shown in Fig. 1c, bacterially-purified GST-USP10 and His-HDAC6 were able to interact with each other under cell-free conditions via a GST pull-down assay, suggesting a direct interaction between USP10 and HDAC6.

Next, we mapped which domain(s) of HDAC6 binds to USP10, and vice versa. As shown in Fig. 1d, e, full-length, two catalytic domains of HDAC6: N-terminal DAC1 and central DAC2, but not C-terminal ZnF-UBP domain, bind to USP10. USP10 domain mapping found a more restricted binding pattern, as besides the full-length, the very C-terminal region (amino acids 700–798) was the only one necessary for the interaction between USP10 and HDAC6 to occur (Fig. 1f, g).

### HDAC6 is a new substrate of USP10

**Fig. 2:**
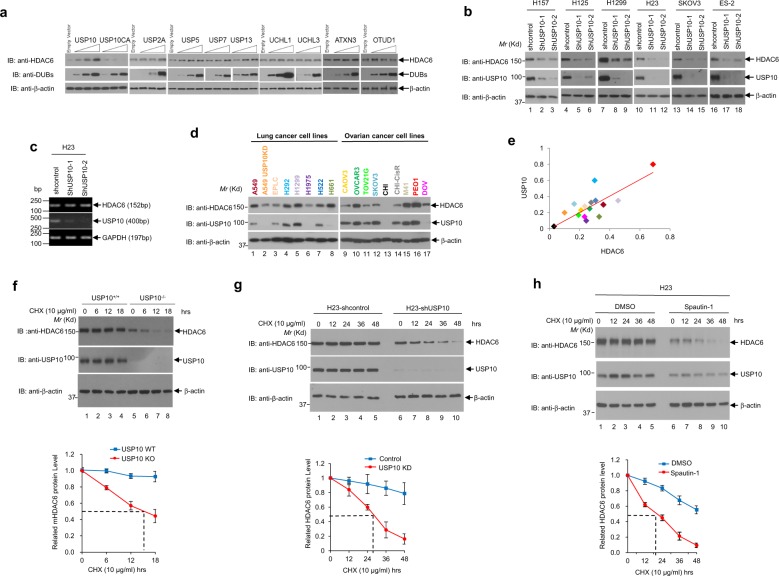
USP10 stabilizes HDAC6. **a** 293T cells were transfected with empty vector, wild-type USP10 (Flag-USP10), catalytically-dead mutant of USP10 (Flag-USP10CA) or other DUBs as indicated. Thirty-six hours post-transfection, cells were harvested. Anti-HDAC6, anti-USP10, anti-DUBs, and anti-β-actin Western blotting analyses were performed. **b** Depletion of USP10 reduces the level of HDAC6. Six lung and ovarian cancer cell lines, H157, H125, H1299, H23, SKOV3, and ES-2, were infected with lentivirus encoding two shRNAs against USP10. The anti-USP10, anti-HDAC6, and anti-β-actin Western blotting analyses were performed as indicated. **c** Depletion of USP10 does not affect the mRNA level of HDAC6 in H23 cells. Total RNAs extracted from shcontrol, shUSP10-1 or shUSP10-2 lentivirus stably infected H23 cells were subjected to RT-PCR for HDAC6, USP10, and GAPDH, as indicated. **d** HDAC6 protein levels are positively correlated with USP10 in a panel of lung and ovarian cancer cell lines. HDAC6, USP10, and β-actin protein levels were obtained by Western blotting analyses of 17 cell lines (A549, A549-USP10KD, EPLC, H292, H1299, H1975, H522, H661, CAOV3, OVCAR3, TOV21G, SKOV3, CHI, CHI-CisR, M41, PEO1, and DOV). **e** HDAC6 and USP10 protein levels were normalized by the level of β-actin and quantified with Image Pro Plus 6.0 software. The correlation analysis was performed by Excel CORREL function and the results were shown as a scatter plot indicating a strong positive correlation coefficient of USP10 and HDAC6 (*r* = 0.78, *p* < 0.01). **f** The half**-**life of HDAC6 is shortened in USP10KO MEFs. Cycloheximide (CHX) was added to USP10 WT and USP10 KO MEFs at the indicated concentration and time intervals. The anti-HDAC6, anti-USP10, and anti-β-actin Western blotting analyses were performed (top panel). The experiments were repeated three times. The HDAC6 expression was quantified and a plot showing half-life of HDAC6 in USP10 WT and USP10 KO MEFs was drawn (lower panel). **g** The half**-**life of HDAC6 is shortened in USP10KD H23 cells. Half-lives of HDAC6 in H23-shcontrol and H23-shUSP10 cells were performed as **f**. **h** USP10 inhibitor Spautin-1 shortens the half-life of HDAC6. Spautin-1 was added to the H23 cells at the indicated concentrations and time intervals. The anti-HDAC6, anti-USP10, and anti-β-actin Western blotting analyses were performed. Half-life of HDAC6 in DMSO-treated or Spautin-1-treated H23 cells was performed as **f**. For graphs in **f**–**h**, the mean band intensities from three independent experiments as measured by Image-Pro plus 6.0 shows the approximate half-lives in the presence of CHX. The error bars represent the standard deviation.

To determine whether USP10’s enzymatic activity regulates the protein expression of HDAC6, we first overexpressed wild-type or catalytically-dead mutant USP10 in 293T cells. As shown in Fig. 2a (lanes 1–7), only wild-type USP10, but not the catalytically deficient mutant (USP10/C424A), significantly increased the endogenous level of HDAC6, indicating that USP10’s deubiquitinase activity is imperative for increasing the protein level of HDAC6. To examine whether other DUBs can also regulate HDAC6, we selected eight cysteine proteases, including four USP family members (USP2A, USP5, USP7, USP13), two UCH family members (UCHL1 and UCHL3), one MJD family member (ATXN3), and one OTU family member (OTUD1). As shown in Fig. 2a (lanes 8–36), none of these DUBs significantly increased the protein level of HDAC6, suggesting that USP10’s role of increasing the level of HDAC6 is specific. To verify the role of USP10 in regulating HDAC6 protein levels, we next depleted USP10 with specific shRNAs and observed a significant decrease of HDAC6 protein expression in four NSCLC cell lines: H157, H125, H1299, and H23, as well as two ovarian cell lines: SKOV3 and ES-2 (Fig. 2b). As a proof-of-concept experiment, we examined the HDAC6 mRNA level in control and two USP10 knockdown H23 cell lines. As shown in Fig. 2c, the mRNA level of HDAC6 was not affected by USP10 knockdown, suggesting that regulation of HDAC6 by USP10 is not through transcription. In an attempt to establish the correlation between USP10 and HDAC6 expression in cancer cell lines, we examined USP10 and HDAC6 protein levels in eight lung cancer lines and nine ovarian cancer cell lines. As shown in Fig. 2d, e, HDAC6 protein levels were found to be strongly and positively correlated with USP10 protein levels (*r* = 0.78, *p* < 0.01) in these cell lines. The above results suggest that USP10 up-regulates HDAC6 protein expression, most likely through deubiquitinating and consequently stabilizing HDAC6. To test this hypothesis, we first examined HDAC6’s half-life by treating USP10 wild-type (WT) and USP10 knockout (KO) mouse embryonic fibroblasts (MEFs) with protein synthesis inhibitor cycloheximide (CHX). As shown in Fig. 2f, CHX treatment rendered a sharper decrease of HDAC6 protein level over the time course, leading a shorter half-life of HDAC6 in USP10-KO MEFs as compared to USP10-WT MEFs. Similarly, knockdown of USP10 in H23 NSCLC cells shortened the half-life of HDAC6 as compared to that in control cells (Fig. 2g). Consistently, direct inhibition of USP10 with its specific inhibitor, Spautin-1^9^, also led to a significant decrease in HDAC6’s protein half-life (Fig. 2h). Overall, our data suggest that USP10 increases HDAC6 protein stability.

**Fig. 3:**
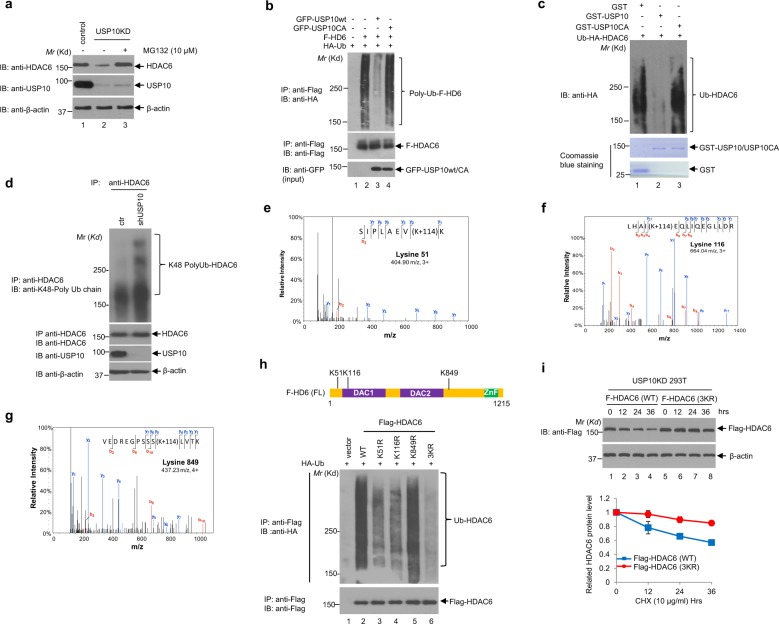
USP10 deubiquitinates HDAC6. **a** USP10 regulates the protein level of HDAC6 *via* the ubiquitin-proteasome pathway. H23 control and H23 USP10 stable knockdown (USP10KD) cells were either left untreated or treated with MG132 for 10 h, then were lysed and subjected to Western blotting analyses as indicated. **b** Wild-type, but not the catalytically-dead mutant of USP10, deubiquitinates HDAC6 in vivo. 293T cells were transfected with the indicated plasmids. The anti-Flag denatured immunoprecipitation was performed followed by anti-HA Western blotting analysis (upper panel). The blot was stripped and reprobed with anti-Flag antibody (middle panel). The anti-GFP Western blotting analysis was performed to show the input of GFP-USP10WT and GFP-USP10CA. **c** Wild-type, but not the catalytically-dead mutant of USP10, deubiquitinates HDAC6 in vitro. Ubiquitinated HA-HDAC6 proteins isolated from 293T cells were pulled down by anti-HA agarose beads, followed by incubation with bacterial purified GST, GST-USP10, or GST-USP10CA proteins as described in the Methods. HDAC6 ubiquitination levels were determined by Western blotting with anti-HA (top panel), and the amount of GST, GST-USP10, and GST-USP10CA proteins were confirmed by coomassie blue staining (bottom two panels). **d** Knockdown of USP10 increases the K48-linked poly-ubiquitination of HDAC6. H1299 cells stably expressing shControl or shUSP10 shRNAs were treated with MG132 (5 µM) overnight. The anti-HDAC6 antibody was used to immunoprecipitate HDAC6 in control and USP10KD cells. Half of the samples were subject to anti-K48 poly-Ub Western blotting analysis; the other half of the samples were subject to anti-HDAC6 Western blotting analysis as indicated. The anti-USP10 and anti-β-actin Western blotting analyses were also performed using total cell lysates. **e–g** Representative MS2 spectra showing putative ubiquitin binding sites Lysines 51, 116, and 849 within HDAC6. Recombinant HDAC6 was immunoprecipitated, separated by SDS-PAGE and digested in-gel with trypsin. Peptides were analyzed by LC-MS/MS. Ubiquitination commonly occurs as the last amino acid of ubiquitin is covalently linked to a lysine residue on the substrate. Since the last three ubiquitin residues are Arg/Gly/Gly, tryptic cleavage of ubiquitinated histidine residues can by identified by Gly/Gly modification (+114). Inset: Fragmentation patterns of *b* and *y* ions show sequence information and localization of the Gly/Gly histidine modification. Also shown are the modified amino acid residue number for HDAC6, m/z and charge state. **h** Lysines 51, 116, 849 are targeted for ubiquitination of HDAC6. Upper panel: The diagram of HDAC6 showing HDAC6 domains and the three ubiquitination sites. Lower panel: HA-Ub was cotransfected with either Flag-HDAC6 wild-type or Flag-HDAC6 Ub site mutants as indicated into 293T cells. Anti-Flag-M2 agarose beads were used to IP Flag-HDAC6. Anti-HA Western blotting analysis was performed to detect the ubiquitination level of HDAC6. **i** Mutation of the three ubiquitination sites (K51, K116, and K849) in HDAC6 prolongs HDAC6’s half-life. USP10 stable knockdown 293T cells were transfected with either Flag-HDAC6 wild-type (WT) or Flag-HDAC6 K51/116/849R (3KR) followed by CHX treatment at indicated time intervals. Anti-Flag and anti-β-actin Western blotting analyses were performed (upper panel). A graph of the mean band intensities from three independent experiments as measured by Image-Pro plus 6.0 shows the approximate half-lives of HDAC6 wild type and the triple site mutant in the presence of CHX. The error bars represent the standard deviation (low panel).

To verify that HDAC6 protein degradation occurs through the ubiquitin-proteasome pathway, the proteasome inhibitor MG132 was added to the USP10-knockdown H23 cells. As shown in Fig. 3a, knockdown of USP10 decreased HDAC6 protein levels as compared to the control H23 cells, while MG132 restored HDAC6 expression in the knockdown cells, indicating that blocking proteasome degradation leads to HDAC6 accumulation. Therefore, USP10 regulates HDAC6 stability through the proteasomal degradation pathway. Next, we tested whether USP10 could deubiquitinate HDAC6. To this end, an in vivo deubiqutination assay was performed. As shown in Fig. 3b, wild-type USP10, but not the catalytically-inactive USP10CA mutant, significantly reduces HDAC6 ubiquitination in 293T cells (comparing lanes 3 and 4 in the top panel). To rule out the possibility that USP10-associated proteins function as DUBs for HDAC6, an in vitro deubiquitination assay was performed using the recombinant wild-type or enzymatically-dead mutant of USP10 (USP10CA) purified from bacteria and ubiquitinated HDAC6 protein as a substrate. Consistent with Fig. 3b, wild-type USP10, but not the catalytically deficient mutant (USP10CA), efficiently removed the poly-ubiquitin chains from HDAC6 (Fig. 3c). To further discern the linkage of the poly-ubiquitin chains on HDAC6 removed by USP10, we took advantage of ubiquitin chain linkage-specific antibodies. As shown in Fig. 3d, the depletion of USP10 in H1299 cells significantly increased the presence of K48-linked polyubiquitin chains in HDAC6. Because attachment of a K48-linked polyubiquitin serves as a signal for ubiquitin-proteasome degradation of the substrate protein^37^, our result confirms the notion that USP10 removes the ubiquitin chain from HDAC6, which in turn stabilizes HDAC6. Taken together, we conclude that USP10 is a DUB for HDAC6.

We next sought to determine the specific ubiquitination sites in HDAC6 from which USP10 removes the polyubiquitin chains. To identify HDAC6 ubiquitination sites, we co-overexpressed HDAC6 and ubiquitin in 293T cells followed by treatment with MG132. The ubiquitinated HDAC6 was immunoprecipitated and resolved on SDS-PAGE, and the ubiquitinated HDAC6 bands were subjected to mass spectrometry analysis. The results revealed that HDAC6 is ubiquitinated at three lysine residues: K51, K116, and K849 (Fig. 3e–g). To ensure that these sites are relevant in vivo, we mutated these three sites individually to arginine to generate K51R, K116R, and K849R mutants. We also mutated all three lysines to arginines to generate one triple mutant, 3KR (K51/116/849R). As shown in Fig. 3h, the ubiquitination levels of the K51R, K116R, and 3KR mutants, but not the K849R mutant, were significantly decreased compared to that of wild-type HDAC6, suggesting that K51 and K116 are the major ubiquitination sites in vivo. To further confirm this result, we transfected the wild-type HDAC6 and the 3KR mutant into USP10 stable knockdown 293T cells. Assessment of the protein half-life of wild-type HDAC6 and 3KR mutant HDAC6 *via* CHX treatment found that the 3KR mutant was much more stable than the wild-type HDAC6 (Fig. 3i). Together, these data indicate that USP10 is an HDAC6 deubiquitinase, which could prolong HDAC6 stability by removing HDAC6’s K48-linked poly-ubiquitin chains at specific lysine residues including K51 and K116. Additionally, to study the function of the 3KR mutant, we overexpressed wild-type and 3KR mutant of HDAC6 in a 293T HDAC6 knockout cell line. We found that ectopic expression of HDAC6 induces cisplatin resistance as compared to the control, which lacks HDAC6 (Fig. S2). We have also found that the 3KR mutant further enhanced cisplatin resistance as compared to the wild-type (Fig. S2). This finding indicates that stabilization of HDAC6 confers cisplatin resistance.

### Depletion or inhibition of USP10 sensitizes NSCLC cells and ovarian cancer cells harboring mutant- or null-p53 to cisplatin

**Fig. 4:**
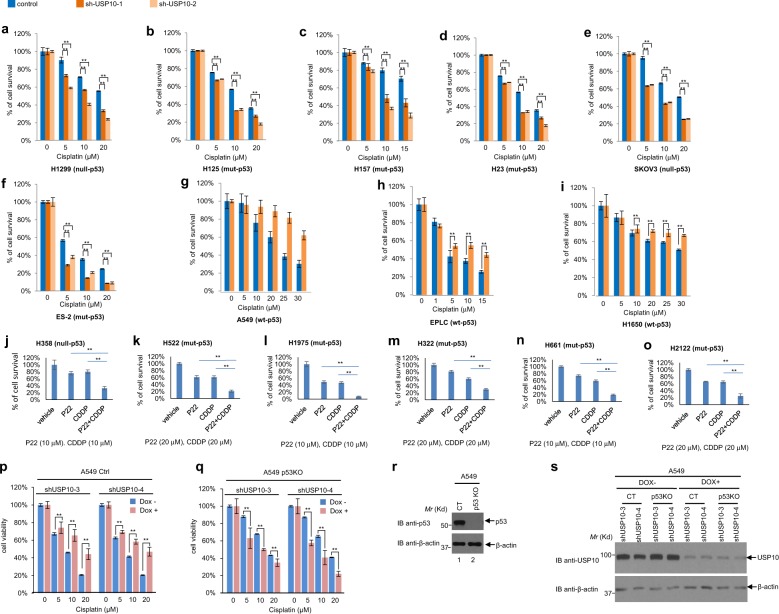
Knockdown of USP10 sensitizes lung and ovarian cancer cells harboring mutant- or null- p53, but not wild-type p53, to cisplatin by MTT assays. **a–i** Indicated control cell line and two USP10 stable knockdown counterparts were subjected to a 3-day MTT assay. The dosage of cisplatin used is indicated. **j–o** Indicated cell lines were treated with vehicle, P22077 (P22), cisplatin (CDDP), or P22 + CDDP and were subjected to a 2-day MTT assay, except **l**, which was subject to a 3-day MTT assay. **p** Doxycycline (Dox)-induced two USP10 knockdowns in A549-control (Ctrl or CT) cell lines: A549-shUSP10-3 and A549-shUSP10-4, were pretreated with 1 μg/ml Doxycycline for 3 days, then were co-treated with cisplatin as indicated concentrations for another 3 days. MTT assays were performed to measure cell viability after treatment. **q** Dox-induced two USP10 knockdowns in A549-p53KO cell lines: A549-p53KO-shUSP10-3, A549-p53KO-shUSP10-4 were pretreated with 1 μg/ml Doxycycline for 3 days, then were co-treated with cisplatin as indicated concentrations for another 3 days. MTT assays were performed to measure cell viability after treatment. **r** Anti-p53 and anti−β-actin Western blotting analyses were performed with A549-(CT) and A549-(p53KO) cells. **s** A549-(CT) and A549-(p53KO) cells were treated with either vehicle or 1 μg/ml Doxycycline for 3 days and whole cell lysate were subjected to Western blot analysis using indicated antibodies. For **a–q**, the error bars represent the standard deviation. Double asterisk indicates *p* < 0.01.

Our previous work has shown that HDAC6 confers cisplatin resistance in NSCLC cell lines^35^. Because USP10 stabilizes HDAC6, we wondered whether USP10 confers cisplatin resistance *via* HDAC6. To test this hypothesis, we depleted USP10 in seven NSCLC and two ovarian cancer cell lines and treated them with either a vehicle or cisplatin to examine cell viability by MTT assays. As shown in Fig. 4a–i, six cell lines harboring null- or mutant-p53 (H1299, H125, H157, H23, SKOV3, and ES-2) were sensitive to cisplatin after USP10 knockdown, whereas those three cell lines harboring wild-type p53 (A549, EPLC, and H1650) were resistant to cisplatin after USP10 knockdown. We next utilized the pharmacological USP10 inhibitor, P22077^14^, to inhibit USP10 in six NSCLC cell lines harboring null- or mutant-p53 (H358, H522, H1975, H322, H661, and H2122) to examine cell viability by MTT assays. As shown in Fig. 4j–o, combination of P22077 and cisplatin treatment significantly reduced cell viability when compared to either P22077 or cisplatin treatment alone. This result corroborates the notion that depletion or inhibition of USP10 sensitizes lung or ovarian cancer cells harboring null- or mutant-p53 to cisplatin.

To determine whether p53 is a determinant for cisplatin sensitivity after USP10 knockdown, we utilized a pair of A549-control and A549-p53 knockout cell lines^38^. Two shRNAs (shUSP10-3 and shUSP10-4) against USP10 were used to deplete USP10 in both cell lines under the control of a doxycycline-inducible system as described in the Methods. As shown in Fig. 4p–q, depletion of USP10 sensitized A549-p53KO cells, but not A549 control cells. Figure 4r–s confirms the p53 and USP10 status of the A549-control and A549-p53 KO cell lines. This result suggests that selectively targeting USP10 in a p53-null background would sensitize cancer cells to cisplatin.

**Fig. 5:**
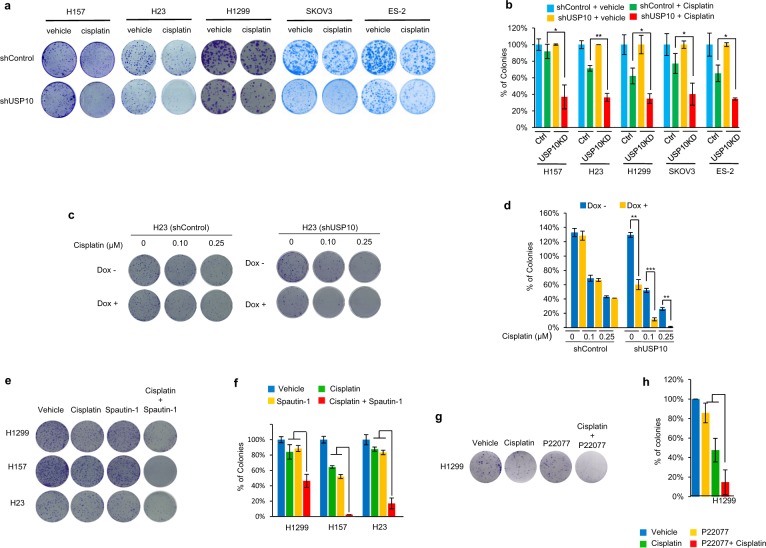
Knockdown of USP10 sensitizes lung and ovarian cancer cells harboring mutant- or null-p53 to cisplatin by colony formation assays. **a**, **b** Knockdown of USP10 sensitizes three lung and two ovarian cancer cell lines to cisplatin by colony formation assays. Control and USP10 stable knockdown H157, H23, H1299, SKOV3, and ES-2 cells were treated with cisplatin at the various concentrations (H157, 2 μM; H23, 0.5 μM; H1299, 1 μM; SKOV3 and ES-2, 0.3 μM) for 7–14 days. Colony formation assay was performed as described in the Methods. Colonies were visualized by crystal violet staining (**a**). Colonies numbers were quantified with OpenCFU software. The percentage of colony formation of indicated cells are shown in (**b**). **c**, **d** Inducible knockdown USP10 sensitizes H23 cells to cisplatin by colony formation assays. H23 control and stable inducible USP10 knockdown pool cells were plated at 100 cells/well into six-well dishes and cultured with or without Dox and then treated with cisplatin as indicated for ~14 days. Colonies were fixed, stained with methanol/crystal violet dye, and images captured (**c**). Colony numbers were counted by OpenCFU software (**d**). **e**, **f** Inhibition of USP10 by spautin 1 sensitizes H1299, H157 and H23 cells to cisplatin in colony formation assays. H1299, H157, and H23 cells were treated with various concentrations of cisplatin (0.5 μM for H1299 and H157; 0.1 μM for H23), USP10 inhibitor spautin-1 (1 μM for H1299, 2 μM for H157, and 0.5 μM for H23), or a combination of cisplatin and spautin-1 for 7–14 days. Colonies were fixed, stained with methanol/crystal violet dye, and images captured (**e**). The percentages of colony formation of the indicated cell lines are shown in (**f**). **g**, **h** USP10 inhibitor P22077 sensitizes H1299 cells to cisplatin in colony formation assays. Colony formation assays for H1299 were performed as **e**, **f**, except that spautin-1 was replaced by P22077 (2 μM) and that cisplatin concentration was 0.5 μM. For all the panels, each assay was performed in triplicate. The error bar represents standard deviation. *p < 0.05, **p < 0.01, ***p < 0.001.

**Fig. 6:**
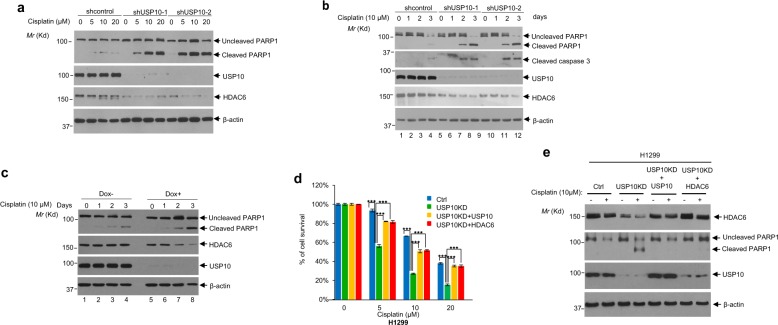
Depletion of USP10 increases apoptosis in NSCLC cells upon cisplatin treatment; USP10 confers cisplatin resistance via HDAC6. **a** Knockdown of USP10 increases apoptosis upon treatment of cisplatin. H157 cells infected with lentiviruses containing empty vector, shUSP10-1, or shUSP10-2 were treated with cisplatin at the indicated concentrations for 3 days. Cells were lysed, then anti-PARP1, anti-USP10, anti-β-actin and anti-HDAC6 western blotting analyses were performed. **b** H23 control and USP10 stable knockdown cells were treated with 10 µM cisplatin at the indicated times. Cells were lysed and the anti-PARP1, anti-cleaved caspase 3, and anti-β-actin Western blotting analyses were performed. **c** Treatment of Dox-induced USP10 knockdown H23 cells with cisplatin increases PARP1 cleavage. H23 cells were pretreated with 1 µg/ml Dox for ~2 days. Cells were then treated with 10 μM cisplatin for the indicated times. Cell lysates were collected for western blotting analyses. **d**, **e** Overexpression of USP10 or HDAC6 in USP10-knockdown H1299 cells rescues USP10 knockdown-induced growth reduction and apoptosis upon cisplatin treatment. H1299 control and H1299 USP10 stable knockdown cells, or H1299 USP10 KD cells reintroduced with either USP10 expression plasmid or HDAC6 expression plasmid at each indicated combination, were treated with the indicated concentration of cisplatin for the 3 days. MTT assays were performed in **d**. Anti-PARP1 and anti-β-actin Western blotting analyses were performed in **e**. The error bar represents standard deviation. Triple asterisk indicates *p* < 0.001.

We next examined the impact of USP10 depletion and cisplatin treatment on long-term cell survival. To this end, we performed colony formation assays using three pairs of control and USP10 stable knockdown NSCLC cell lines (H157, H23 and H1299) and two pairs of control and USP10 stable knockdown ovarian cancer cell lines (SKOV3 and ES-2), all of which harbor null- or mutant-p53. As shown in Fig. 5a–b, **s**table depletion of USP10 greatly sensitized all cell lines to cisplatin. To further confirm our results and avoid variation between individual stable clones, we employed an inducible lentiviral pTRIPZ-Tet-On vector system as described in the Methods, in which expression of shRNA against USP10 is induced in the presence of doxycycline. As shown in Fig. 6c, doxycycline induction of USP10 shRNA (Dox+), but not the vehicle (Dox−), led to a significant reduction of USP10 in H23 cells. Cells were then plated in the presence or absence of doxycycline, and the results show that USP10 inducible knockdown led to reduced colony formation capacity, a phenotype that was exacerbated after cisplatin treatment (Fig. 5c–d). To determine whether inhibition of USP10 activity will achieve similar outcomes to depletion of USP10, we treated three NSCLC cell lines, H1299, H157, and H23, with the USP10 inhibitor Spautin-1^9^, and H1299 with an additional USP10 inhibitor P22077^14^. As shown in Fig. 5e-h, cisplatin in combination with either USP10 inhibitor suppressed colony growth greater than either cisplatin or USP10 inhibitor alone, suggesting that targeting the activity of USP10 will sensitize NSCLC cells to cisplatin.

Subsequently, to determine whether the USP10 knockdown-induced increase in cisplatin sensitivity is due to apoptosis, we examined PARP-1 cleavage in two NSCLS cell lines: H157 and H23. As expected, the level of cleaved PARP-1 was elevated in two USP10 stable knockdown H157 cell lines, H157-shUSP10-1 and H157-shUSP10-2, compared with that in control knockdown cells (H157-shcontrol) in a cisplatin dose-dependent manner (Fig. 6a). Consistent with the above results, the levels of cleaved PARP-1 and cleaved caspase 3 were significantly increased in two USP10 knockdown H23 cell lines, H23-shUSP10-1 and H23-shUSP10-2, post-treatment with cisplatin in a time-dependent manner compared to control cells, H23-shcontrol (Fig. 6b). We also verified the above results using a dox-inducible USP10 knockdown H23 cell line. As shown in Fig. 6c, upon induction of USP10 knockdown by doxycycline and cisplatin treatment, the levels of cleaved PARP-1 were increased compared to the non-dox induced cells (lanes 5–8 vs lanes 1–4, top panel), suggesting that temporary depletion of USP10 renders cisplatin sensitivity. HDAC6 protein levels were also decreased in USP10 stable and inducible knockdown cell lines, as expected. Therefore, USP10 may decrease cisplatin sensitivity and serve an oncogenic role through HDAC6. To this end, we reintroduced either HDAC6 or USP10 into USP10-depleted H1299 cells. As shown in Fig. 6d–e, overexpression of either HDAC6 or USP10 in USP10 knockdown cells significantly increased cell survival post-cisplatin treatment (Fig. 6d) and reduced apoptosis as shown by the level of cleaved PARP-1 (Fig. 6e). Therefore, these results indicate that a USP10-HDAC6 axis exists, and that this axis plays an essential role in deciding cisplatin sensitivity and oncogenic functions of USP10 in the absence of wild-type p53.

### Depletion or inhibition of USP10 reduces xenograft growth and sensitizes xenografts to cisplatin

**Fig. 7:**
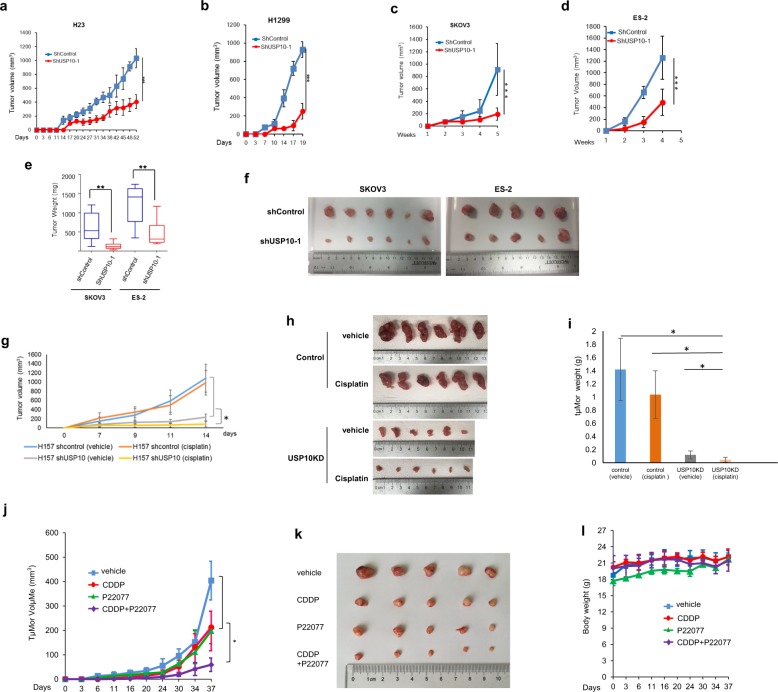
Knockdown or inhibition of USP10 suppresses lung and ovarian cancer xenograft growth and sensitizes xenografts to cisplatin treatment in immune-deficient mice. **a–d** Knockdown of USP10 inhibits H23, H1299, SKOV3, and ES-2 xenograft growth in immune-deficient mice. **a**, **b** The volumes of H23 and H1299 control implants or H23 and H1299 USP10 knockdown implants in SCID mice were measured every 2–3 days as described in the Methods. **c**, **d** The SKOV3 and ES-2 control or SKOV3 and ES-2 USP10 knockdown cells were injected into the nude mice as described in the Methods. The tumor volumes were measured weekly. **e**, **f** The SKOV3 and ES-2 control or SKOV3 and ES-2 USP10 knockdown xenografts were weighted (**e**) and the tumors’ images were taken as shown in **f**. For SKOV3, *n* = 6; for ES-2, *n* = 5. **g–i** Knockdown of USP10 sensitizes the H157 xenografts to cisplatin. H157-control and H157-USP10KD tumors were treated *i.v.* with either vehicle or cisplatin (at 2 mg/kg), starting on day 1 on a Q3dx5 schedule for a total dose of 10 mg/kg. Mice were euthanized 15 days post-implantation. Tumor volumes were shown in **g**. Photos of the tumors were shown in **h**. Tumor weight was quantified in bar graphs shown in **i**. **j** Inhibition of USP10 reduces H1299 tumor growth and sensitizes H1299 xenografts to cisplatin in nude mice. The growth curve of H1299 xenograft tumors treated with vehicle, cisplatin (3 mg/kg, intravenous (*i.v.*) injection), USP10 inhibitor P22077 (15 mg/kg, intraperitoneal (*i.p.*) injection) and cisplatin plus P22077 was shown. Tumor volumes were calculated as above; *n* = 5 per group. **k** Tumor images of H1299 xenografts. **l** Body weight of mice used in **j** was measured during the treatment. * *p* < 0.05, **p < 0.01, ***p < 0.001.

The data presented up to this point prompted us to test whether depletion of USP10 in cancers lacking wild-type p53 decreases tumorigenesis and increases chemosensitivity in vivo. To investigate the effect of USP10 knockdown on tumor growth in vivo, we inoculated four pairs of control and USP10 knockdown cancer cells, H23, H1299, SKOV3, and ES-2 in immune-deficient mice and measured xenograft growth in a time course. As shown in Fig. 7a–d, ablation of USP10 significantly reduced tumor size in all four cell lines tested. Consistently, tumor weight was considerably decreased in USP10 knockdown SKOV3 and ES-2 xenografts (Fig. 7e, f). These results suggest that depletion of USP10 decreases tumor growth in vivo.

We then set out to determine whether knockdown of USP10 sensitizes lung cancer xenografts to cisplatin. To this end, we chose to use a p53-mutant NSCLC H157 cell line for this xenograft study. We used two cisplatin treatment schemes: 3 mg/kg on a Q4dx4 schedule (total dose:12 mg/kg) or 2 mg/kg on a Q3dx5 schedule (total dose:10 mg/kg) (Table S2). The results of the second treatment scheme were shown in Fig. 7g–i. Knockdown of USP10 drastically reduced xenograft growth, and in combination with cisplatin treatment almost completely abolished tumor initiation. As shown in Fig. 7g–i, USP10 knockdown in xenografts sharply reduced both tumor volume (Fig. 7g) and tumor weight (Fig. 7i). Moreover, tumor growth inhibition shown as T/C% (treatment/control ratio) in USP10 knockdown xenograft is 45%, while the one in control xenograft is 95% (Table S2). However, the first treatment scheme did not work as pronounced as the second one. The T/C% in USP10 knockdown xenografts is 62%, which the one in control xenografts is 82% (Table S2), indicating that both the dose and schedule of cisplatin treatment are important for the outcomes. Additionally, to visually show that USP10 knockdown xenografts were more sensitive to cisplatin, we set the volumes of USP10 knockdown xenografts treated with vehicle as 100% and plotted a bar graph (Fig. S3). We also obtained similar results in p53-null NSCLC H1299 xenografts as those in H157 xenografts (Fig. S5). Overall, our in vivo mouse model experiments suggest that depleting USP10 sensitizes the tumors harboring p53-mutant or p53-null to cisplatin. In addition, we also observed that depletion of USP10 significantly decreased the xenografts growth of H1299, SKOV3 (p53-null) as well as H23, ES-2 and H157 (p53-mutant), but not A549 (p53-wild-type) (Figs. 7 and S4), suggesting that p53 is a determinant for growth in vivo mediated by USP10.

We next evaluated the potential therapeutic benefit of USP10 inhibitors on murine xenograft tumors. To this end, we tested the USP10 inhibitor P22077 in vivo, using the p53-deficient NSCLC H1299 cell line. As shown in Fig. 7j, k, single agent treatments of cisplatin or P22077 alone led to significant inhibition of tumor growth compared with the untreated control, whereas P22077 combined with cisplatin showed the most effective inhibition of tumor growth. No weight loss was observed in any of the mice across all treatment groups (Fig. 7l), suggesting that the dosage for P22077 alone or in combination with cisplatin was not toxic to mice. These results indicate that the combination of cisplatin and USP10 inhibition by P22077 exhibits a more potent inhibition of tumor growth than either single agent treatment, and suggest a novel strategy of combining USP10 inhibition with cisplatin for treatment of NSCLC.

### Both USP10 and HDAC6 are highly expressed in lung and ovarian cancers; high levels of USP10 correlate with shorter overall survival in NSCLC patients treated with platinum

**Fig. 8:**
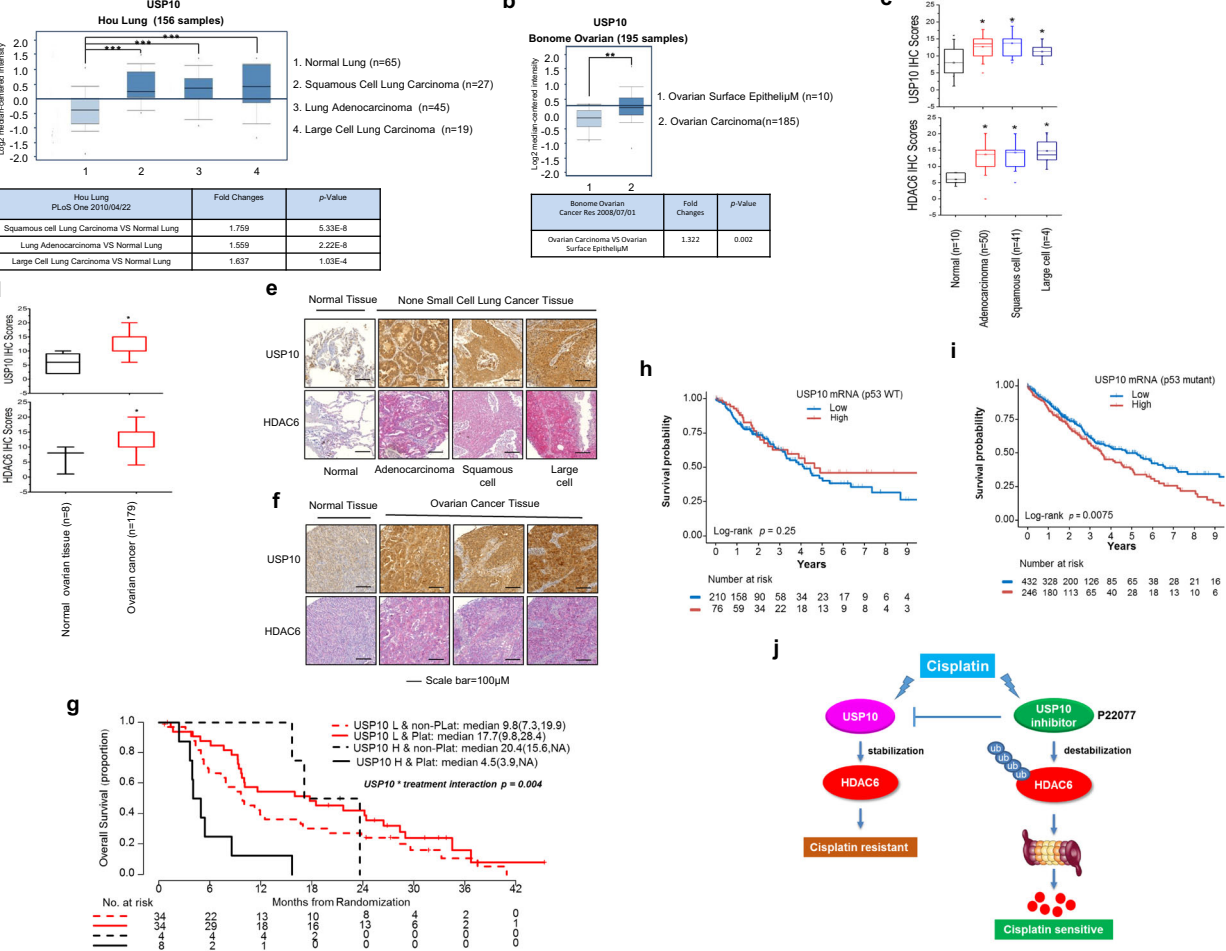
Both mRNA and protein levels of USP10 are increased in lung and ovarian cancer patient samples; high USP10 mRNA level correlates with poor overall survival of advanced NSCLC patients treated with platinum. **a** The *USP10* mRNA expression was elevated in a cohort of NSCLC patients. The box plots were derived from the *USP10* gene expression data in Oncomine. The reference, fold change, *p* value, and sample description were indicated in the table below. **b** The *USP10* mRNA expression was elevated in a cohort of ovarian cancer patients. The box plots were derived from the *USP10* gene expression data in Oncomine. The reference, fold change, *p* value, and sample description were indicated in the table below. **c** The protein levels of USP10 and HDAC6 were elevated in NSCLC patients. The anti-USP10 and anti-HDAC6 IHC staining was performed with a lung cancer TMA (US Biomax. Inc.). The levels of USP10 and HDAC6 were indicated by IHC scores as described in the Methods. **d** The protein levels of USP10 and HDAC6 were elevated in ovarian cancer patients. The anti-USP10 and anti-HDAC6 IHC staining were performed with an ovarian cancer TMA (US Biomax. Inc.). The levels of USP10 and HDAC6 were indicated by IHC scores as described in the Methods. **e** Representative anti-USP10 IHC staining from **c**. **f** Representative anti-USP10 IHC staining from **d. g** The level of USP10 transcript correlates with patient response to platinum treatment, but not to non-platinum treatment. Patient survival according to USP10 high/low status and treatment group. A threshold for USP10 RT-PCR expression was searched using a log-rank test within patients treated with platinum-doublet chemotherapy. The resultant threshold was then used to define the USP10 status of patients treated with non-platinum-doublet chemotherapy. The red and black solid lines indicate OS between low USP10 and high USP10 for patients treated with the platinum doublet. The red and black dotted lines indicate OS between low USP10 and high USP10 for patients treated with the non-platinum doublet. **h**, **i** USP10 expression and p53 status in relation to OS in the TCGA lung cancer data set. **h** The WT p53 cohort were stratified by USP10 high/low expression, or **i** the mutated p53 cohort were stratified by USP10 high/low expression. **j** A working model of USP10 conferring cisplatin resistance by stabilizing HDAC6.

To further validate our findings in human cancer patient samples, we first analyzed changes in USP10 expression in normal or selected cancer tissues by using the online cancer microarray database Oncomine (www.oncomine.org)^39^. The results showed that the mRNA levels of USP10 were significantly overexpressed (*p* < 0.01) in three histological subtypes of NSCLC—lung adenocarcinoma, squamous cell lung carcinoma, and large cell lung carcinoma (Fig. 8a)—and ovarian cancer (Fig. 8b) when compared with normal tissues.

In the aforementioned studies, we had shown a strong positive correlation between USP10 and HDAC6 in seventeen lung and ovarian cancer cell lines (Fig. 2d, e). To further confirm this expression pattern and the relationship between USP10 and HDAC6 in clinical tissue specimens, we obtained high-density lung and ovarian cancer tissue microarrays from US Biomax, Inc. (BC041115 and OV2001) (Fig. S6). Immunohistochemistry (IHC) staining was performed to assess USP10 and HDAC6 expression levels in these patient samples. All tumor section slides were stained by the H. Lee Moffitt Cancer Institute Pathology core facility and were quantified by a pathologist. Scoring was based on intensity, distribution, and subcellular localization as described in the Methods. The results showed that both USP10 and HDAC6 expression were significantly (*p* < 0.01) increased in cancer patient samples compared to normal tissues (Fig. 8c–f). Furthermore, as shown in Table S3, Pearson coefficients for USP10 and HDAC6 expression were ~0.43 and ~0.37 in lung cancer and ovarian cancer, respectively, suggesting a modest positive correlation between expression of USP10 and HDAC6.

Next, we examined whether the levels of USP10 are predictive for platinum response in NSCLC patients. We had previously conducted a randomized international Phase III trial of ERCC1 and RRM1 expression-based chemotherapy versus unselected gemcitabine/carboplatin-based chemotherapy in advanced NSCLC^40^. Characteristics of patients used to investigate USP10 expression are listed in Table S4, and no significant differences between the treatment arms were found as evidenced by all the *p* values being greater than 0.05. There are also no significant differences between USP10 high/low status and a patient’s baseline characteristics either; as shown in Table S5, all the *p* values are greater than 0.05. Importantly, we found a significant interaction between USP10 levels and platinum treatment efficacy. As shown in Fig. 8g, high mRNA levels of USP10 adversely impact overall survival in a cohort of 42 patients treated with platinum. Overall, USP10 is a potential predictive biomarker with a *p* value of 0.004 for treatment-marker interaction in the Cox model adjusted for age, sex, histology, stage, and smoking status.

To examine the impact of USP10 expression in p53 mutant NSCLC patients, we utilized a cohort of 964 patients from TCGA (The Cancer Genome Atlas). As shown in Fig. 8h, i, high levels of USP10 mRNA correlate with lower OS in the p53 mutant, but not in the p53 wild-type cohort, suggesting that USP10 is a potential target in a subset of NSCLC with p53 mutations.

Collectively, our studies characterize USP10’s role as a deubiquitinase for HDAC6. Depletion or inhibition of USP10 significantly decreases HDAC6 protein levels, inhibits tumor growth, and increases p53-mutant/null cancer cell sensitivity to cisplatin in a xenograft mouse model. Rescuing HDAC6 or USP10 in USP10 knockdown cancer cells yielded a high survival rate and less PARP-1 cleavage. Moreover, review of high-density TMAs showed both USP10 and HDAC6 overexpression in ovarian and lung cancer patient samples and a positive correlation between their expression. USP10 expression is associated with OS in mutant p53 NSCLC patients. Overall, our data suggest targeting the USP10-HDAC6 axis in NSCLC lacking wild-type p53 (Fig. 8j).

## Discussion

We previously showed that HDAC6 regulates 6-thioguanine (6-TG) sensitivity via modulating the level of MSH2^33^. We also showed that USP10 stabilizes MSH2 to regulate 6-TG sensitivity^41^. So, HDAC6 and USP10 exert opposing effect on MSH2: HDAC6 promotes degradation of MSH2, while USP10 promotes stabilization of MSH2. In this current study, we describe that USP10 stabilizes HDAC6 to promote resistance to cisplatin. This is consistent with our previous finding that HDAC6 promotes resistance to 6-TG by downregulating MSH2^33^. However, it seems to contradict the finding that USP10 promotes sensitivity to 6-TG by stabilizing MSH2^41^. When we planned to continue our 2016 study^41^, we noticed that USP10 only promotes sensitivity to 6-TG in A549, a wild-type p53 cell line, but not in H1299, a null-p53 cell line. This finding provided the rationale for us to pursue our current study—to characterize the role of USP10 in cisplatin resistance in a null- or mutant-p53 background.

Our previously published data have shown that targeting HDAC6 (i.e., depletion/inhibition of HDAC6) could sensitize NSCLC cell lines to cisplatin regardless of their p53 status^35^ (and data not shown). We have now shown that targeting USP10 only sensitizes NSCLC cell lines without wild-type p53. Based on the above data, we have proposed two following hypotheses. We first hypothesize that in a wild-type p53 background, depletion of USP10 leads to the reduction of HDAC6 as well as affecting an unknown factor, which counteracts the effect of HDAC6 reduction. Therefore, the level of HDAC6 is not indicative of cisplatin sensitivity in a wild-type p53 background when USP10 is targeted. We also hypothesize that in the absence of p53, USP10 stabilizes HDAC6, leading to cisplatin resistance. Our preliminary data indicates that p53 strongly interrupts the USP10-HDAC6 co-precipitation (data not shown), suggesting that p53 competes with HDAC6 for binding to USP10. In other words, in the presence of p53, USP10 largely binds p53 and stabilizes p53 under the stress conditions. Thus, depletion of USP10 would cause a decrease in p53 levels and lead to cisplatin resistance. In the null-p53 or mutant-p53 background, USP10 may largely bind to HDAC6. However, further experiments would be needed to test both hypotheses.

## Methods

### Cell culture and transfection

HEK293T, SKOV3, ES-2, MEFs were grown in Dulbecco’s Modified Eagle Medium (DMEM) with 10% fetal bovine serum (FBS) and 1% penicillin/streptomycin. A549, EPLC, H292, H1299, H1975, H522, H661, H125, H157, H322, H23, H1650, H358, and H2122 were grown in RPMI 1640 with 10% FBS and 1% penicillin/streptomycin. All cells lines were maintained at 37 °C in a humidified atmosphere of 5% CO_2_. The pellets of a panel of ovarian cancer cell lines, CAOV3, OVCAR3, TOV21G, CHI, CHI-CisR, M41, PEO1 and DOV, were kindly provided by Dr. Johnathan M. Lancaster and Dr. Douglas Marchion from H. Lee Moffitt Cancer Center. Transient transfections were carried out with Lipofectamine 2000 (Invitrogen) or polyethyleneimine (PEI). The USP10 knockdown cell lines were established either with the MISSION® shRNA system (Sigma) or TRIPZ inducible Lentiviral shRNA (GE Dharmacon). Lentiviruses were produced using a standard protocol (http://www.addgene.org/tools/protocols/plko/). USP10 knockout MEFs were generated in Dr. Zhenkun Lou’s laboratory at Mayo Clinic (unpublished data). A549 control and p53KO cells were described in Heyza et al.^38^. All cell lines were purchased from ATCC. Otherwise they were described as the above.

### Plasmids, antibodies, and reagents

The expression plasmids of HA-USP10 and Flag-USP10, Flag-USP10 (C424A) mutant and USP10 deletions were described in Zhang et al.^41^. Expression plasmids of HA-HDAC6 and HA-tagged ubiquitin were described in Zhang et al.^33^. Flag-HDAC6, Flag-HDAC6 (1-503), Flag-HDAC6(486-834), and F-HDAC6(835-1215) were generated by PCR using p3XFlag-CMV10 (Sigma) as a vector. To generate GST-USP10, USP10 cDNA fragments was produced by PCR with a full-length USP10 cDNA as a template as well as two primers, USP10 forward: 5′-CCCTCGAGATGGCCCTCCACAGCCC-3′ containing the XhoI site (underlined) and USP10 reverse: 5′-ATTTGCGGCCGCTTACAGCAGGTCCACTCGGC-3′ containing the NotI site (underlined). The resulting USP10 PCR fragment was cut with XhoI and NotI and was then inserted into the pGEX-4T-1 vector between XhoI and NotI sites. To generate His-HDAC6, HDAC6 cDNA was produced by PCR with full-length cDNA HDAC6 as a template as well as two primers, HDAC6 Forward: 5′-GGAAGATCTGATGACCTCAACCGGCCAGGATTC-3′ containing the BglII site (underlined) and HDAC6 Reverse: 5′-AAATATGCGGCCGCAGTAGTGTGGGTGGGGCATATCCTCC-3′ containing the NotI site (underlined). The resulting HDAC6 PCR fragment was cut with BglII and NotI and inserted into the pET-30a vector between BglII and NotI sites. F-USP10 and F-USP10CA plasmids were described in Yuan et al.^5^ and Zhang et al.^41^ respectively. GFP-USP10 and GFP-USP10CA plasmids were obtained from Dr. Zhenkun Lou from Mayo Clinic. USP2A, USP5, USP7, USP13, UCHL1, UCHL3, ATXN3, OTUD1 expression plasmids were gifts from Dr. Jia Fang from H. Lee Moffitt Cancer Center and Research Institute. USP10 knockdown shRNA plasmid was obtained from Sigma (TRC Human USP10 shRNA, Clone ID: TRCN0000007430 and TRCN0000007431). USP10 inducible knockdown plasmids were purchased from GE Dharmacon: TRIPZ Inducible Lentiviral Human USP10 shRNAs, which contain five pairs of shRNA: V2THS_65459, V2THS_65461, V3THS_361007, V3THS_361008(shUSP10-03), V3THS_361009(shUSP10-04).

The Flag-HDAC6 mutants K51R, K116R, K849R, and 3KR (K51/K116/K849R) plasmids were generated by mutating lysine into arginine using the Flag-HDAC6 vector as a template via PCR with the QuickChange Site-Directed Mutagenesis Kit (Agilent Technologies). Primers for these constructions are as follows: K51R-Forward: 5′-TCATTTTGCCTTTCTTCCTTACCTCCGCTAGATTGGG-3′; K51R-Reverse: 5′-CCCAATCTAGCGGAGGTAAGGAAGAAAGGCAAAATGA-3′; K116R-Forward: 5′-TCAGTTGCTCCCTGATGGCATGGAGCCGC-3′; K116R-Reverse: 5′-GCGGCTCCATGCCATCAGGGAGCAACTGA-3′; K849R-Forward: 5′-CTTCTTGGTGACCAACCTAGAACTGGAGGGTCC-3′; K849R-Reverse: 5′- GGACCCTCCAGTTCTAGGTTGGTCACCAAGAAG-3′. The PCR products were inserted into the NotI and XbaI sites of the p3XFlag-CMV10 vector from Sigma to generate Flag-HDAC6(K51R), Flag-HDAC6(K116R), Flag-HDAC6(K849R) and Flag-HDAC6(3KR). The backbone of Flag-HDAC6 is also p3XFlag-CMV10 (Sigma).

Anti-Flag M2 Affinity Agarose Gel, anti-HA Agarose, Flag peptide, cycloheximide, cisplatin, spautin-1, MG132, Thiazolyl Blue Tetrazolium Bromide (for MTT assays) and doxycycline were purchased from Sigma. P22077 was purchased from Selleckchem.com. ECL western blotting substrates were obtained from ThermoFisher Scientific (Catalog number: 32106 and 34076).

Anti-USP10 antibody (ab72486), anti-Ubiquitin (linkage-specific K48) antibody (ab140601), and anti-Ubiquitin (linkage-specific K63) antibody (ab179434) was obtained from Abcam. Anti-HDAC6 (H-300) and anti-HA (Y-11) antibodies were purchased from Santa Cruz Biotechnology, Inc. Anti-HDAC6 (SAB4500012), anti-Flag M2 antibody and anti-Flag-M2 beads were purchased from Sigma. Anti-PARP (46D11) was obtained from Cell Signaling Technology. Horseradish peroxidase (HRP) conjugated secondary antibodies were purchased from Sigma: Rabbit IgG HRP linked whole Ab (NV934V) and Mouse IgG HRP linked whole Ab (NA931V).

### MTT assay

Five thousand cells were seeded in quintuplicate in 96-well plates. The drugs were added 24 h after seeding, while vehicle was added as the control. At the indicated days, cells were incubated with 3-(4, 5-dimethylthiszol-x-yl)-2, 5-diphenyltetrazolium bromide (MTT) (Sigma) solution for 2 hours, then supplemented with 150 μl of DMSO and shaken for 10 min at RT. The absorbance of exposed cultures was measured using a multi-well spectrophotometer (MiniMax^TM^ 300 Imaging Cytometer) at 490 nm. The results were presented as a percentage of absorbance relative to vehicle control cultures.

### Immunoprecipitation and Immunoblotting

Cells were lysed on ice in lysis buffer (20 mM Tris-HCl [pH 7.5], 150 mM NaCl, 1 mM Na_2_EDTA, 1 mM EGTA, 1% Triton X-100, 10% glycerol and protease inhibitor cocktail). For Co-immunoprecipitation (Co-IP), lysates were incubated with anti-Flag M2 agarose or anti-HA agarose for about 4 h at 4 °C. For endogenous immunoprecipitation, lysates were incubated with protein A or protein G agarose (Invitrogen) for 1 h of pre-clearing before incubation with primary antibodies for 12 hr at 4 °C, then incubated with Protein A or Protein G beads for another one hour. Immunocomplexes were then collected, washed three times in lysis buffer and resolved on SDS-PAGE. For immunoblotting, samples were transferred to nitrocellulose membranes that were then probed with antibodies. Proteins recognized by the antibodies were detected using Thermo Scientific ECL substrate kit.

### RT-PCR assay

Reverse transcriptase-polymerase chain reaction (RT-PCR) assays were performed to measure the expression of mRNA. Cells were washed at least twice with PBS and immediately lysed in Trizol^®^ (AMBION, Catalog number: 15596026). Total RNA was then isolated. Subsequently, 1 μg of RNA was reverse-transcribed using the WarmStart^®^ RTx reverse transcriptase (New England BioLabs, M0380L) and random primer mix (New England BioLabs, S1330S) according to the typical cDNA synthesis protocol. PCR reactions were performed with Taq 2X Master Mix (New England BioLabs, M0270L). The thermocycler conditions for human HDAC6 PCR product were as follows: 95°C 30 sec for 1 cycle; 95 °C 30 s, 55 °C 60 s, and 68 °C 1 min for 40 cycles; final extension 68 °C 5 min for 1 cycle. The same thermocycler conditions were used for human USP10 and GAPDH, except that 30 cycles were used for USP10 and 25 cycles for GAPDH. The PCR primers were listed as follows: HDAC6-forward: 5′- TCAGGTCTACTGTGGTCGTT-3′; HDAC6-reverse: 5′-TCTTCACATCTAGGAGAGCC-3′; USP10-forward: 5′- ATGATTCTAAGCCCT CTGCCTCCT-3′; USP10-reverse: 5′- ATTCATGAGCCGAACAAAGCTATC-3′; GAPDH-forward: 5′-GGAGCGAGATCCCTCCAAAAT-3′; GAPDH-reverse: 5′-GGCTGTTGTCATACTTCTCATGG-3.′ The PCR products were then loaded onto agarose gel with ethidium bromide. After gel electrophoresis, the PCR products were visualized under the UV light.

### Identification of ubiquitination sites in HDAC6

Immunoprecipitated HDAC6 protein was resolved on a 7% acrylamide gel by SDS-PAGE and stained with Coomassie blue. An area of the gel lane where ubiquitinated HDAC6 was expected to migrate was excised. Proteins were reduced, alkylated and digested in-gel with trypsin. Eluted peptides were separated by C18 reverse phase chromatography with an EASY-nLC 1000 system (Thermo) and analyzed in a Q Exactive Hybrid Quadrupole-Orbitrap mass spectrometer (Thermo). Peptide-to-spectrum matches (PSMs) were made using the Sequest algorithm within Proteome Discoverer (Thermo; ver 2.1) compared to human forward and reverse protein databases (downloaded from Uniprot on 07-04-2016; 20,154 sequences). Parent and fragment ion tolerances were 10 ppm and 0.02 Da, respectively. Carbamidomethylation of Cys was included as a static modification, and deamidation of Asn/Gln, oxidation of Met, and Gly/Gly on His were included as dynamic modifications in the search, along with up to two missed tryptic cleavages. Results were imported into Scaffold (Proteome Software; ver 4.8) and a subset database was reanalyzed using X! Tandem. Final peptide identifications were determined at ≤1% false discovery rate (FDR).

### In vivo Ubiquitination assay

Flag-HDAC6 was co-transfected with HA-Ub, GFP-USP10, or GFP-USP10C424A mutant into 293 T cells. In all, 36 h post-transfection, cells were harvested under denaturing conditions. 0.1 ml denaturing cell lysis buffer (50 mM Tris [pH 7.5], 1% SDS) was added to each sample, and samples were boiled for 10 min. Then, samples were further diluted with 0.9 ml lysis buffer (20 mM Tris [pH 7.5], 150 mM NaCl, 1 mM EDTA, 1 mM EGTA, 1% Triton X-100) and rotated at 4 °C for another 20 min. Samples were then centrifuged, and supernatant transferred to a new tube along with Flag-M2 agarose for a 6-h incubation. HDAC6 immune complexes were isolated and HDAC6 ubiquitination was analyzed by anti-HA western blotting.

### In vitro deubiquitination assay

We first prepared ubiquitinated HDAC6 proteins using as the substrate for in vitro deubiquitination assay. Briefly, HEK293T cells were transfected with Flag-tagged HDAC6 with HA-ubiquitin, and were treated with 10 µM MG132 for 12 h. Ubiquitinated Flag-HDAC6 was purified from the cell extracts with Flag-M2 Agarose. After extensive washing with lysis buffer, the bound proteins were eluted with Flag peptides. Then the eluted HDAC6 proteins were used in the in vitro deubiquitination assay. GST-USP10 or GST-USP10CA mutant was expressed in the *E. coli* strain BL21. After induction with 0.1 mM Isopropyl β-D-1-thiogalactopyranoside (IPTG) for 2 h at 37°C, cells were lysed, and GST-USP10 or GST-USP10CA was purified with glutathione-Sepharose-4B beads and eluted with PBS containing 10 mM l-glutathione (Sigma). An in vitro deubiquitination reaction was performed as described previously^41^ with minor modifications: briefly, eluted ubiquitinated Flag-HDAC6 was incubated with purified GST-USP10 or GST-USP10CA in deubiquitination buffer (50 mM Tris-HCl [pH8.0], 50 mM NaCl, 1 mM EDTA, 10 mM dithiothreitol and 5% glycerol) for 2 h at 37 °C. After the reaction, HDAC6 was immunoprecipitated with the anti-Flag antibody. The beads were washed with deubiquitylation buffer, and the bound proteins were eluted by boiling in 1x SDS loading buffer and subjected to immunoblotting with anti-HA antibodies.

### Establishment of USP10 stable or inducible knockdown cell lines

To generate the USP10 stable knockdown cell lines, two pairs of shRNA against USP10 (5′-GCCTCTCTTTAGTGGCTCTTT-3′ and 5′-CCTATGTGGAAACTAAGTA-3′) were used to generate lentiviruses. About 24 h after viral infection, cells were split into duplicate plates containing 1 µg/ml puromycin. Puromycin was replenished every 3–5 days to maintain a sufficient level of selection pressure. The well-isolated single clones were transferred into 24-well plates. The knockdown effect was verified by western blotting analysis using the anti-USP10 antibody. To generate USP10 inducible knockdown cell lines, two pairs of shRNA against USP10 (shUSP10-03 and shUSP10-04) in the inducible lentiviral pTRIPZ-Tet-On vector system (GE Healthcare) were used to generate lentivirus and infected cells in a similar way as the above. After puromycin selection, cells were verified by fluorescence microscopy after doxycycline induction (TRIPZ inducible shRNA vector contains a TurboRFP fragment, so red fluorescence can be used as a readout for the presence of USP10 shRNA). Pooled cells, but no single clones, were selected and used for the experiments. We used inducible lentiviral pTRIPZ-Tet-On system to knockdown A549 (CT) and A549 (p53KO). Instead of using puromycin selection, we employed flow cytometry analysis to select RFP-positive cells to establish USP10 inducible knockdown in both A549 (CT) and A549 (p53KO) cell lines. The USP10 inducible knockdown pool was established in H23 cells using the same method as described above. The shUSP10-3 was used to knockdown USP10.

### GST pull-down assay

GST and His fusion proteins were purified as described^33^. Briefly, BL21 cells harboring the GST or various GST and His recombinant expression plasmids were grown to log phase and induced with 0.1 mM IPTG for two hrs at 37°C. Bacteria were collected and re-suspended in lysis buffer and sonicated. Solubilized proteins were recovered by centrifugation and incubated with glutathione-agarose beads or Ni-NTA beads for 4 h at 4 °C, and washed several times with ice-cold PBS. The resulting bead-bound proteins were eluted with the elution buffer. Then, the eluted proteins were subjected to dialysis against PBS overnight at the 4^o^C.

For in vitro binding assays, GST-HDAC6 or GST was mixed with His-USP10 in GST pull-down buffer (20 mM Tris-HCl [pH 7.5], 150 mM NaCl, 0.1% Triton X-100, 1 mM DTT, 10% glycerol) at 4 °C overnight. Then glutathione sepharose was added and samples were rotated at 4 °C for 1 h. After washing three times, the bound proteins were separated on SDS-PAGE followed by immunoblotting analysis.

### Colony formation assay

Cells were seeded in six-well plates (500 cells per well) and incubated overnight at 37 °C allowing cells to attach to the dishes. Cells were cultured in the absence or presence of drugs. After ~1–2 weeks, cells were washed with PBS and fixed with crystal violet staining buffer (PBS with 0.1%w/v crystal violet, 1% formaldehyde, 1% methanol) for 20 min at room temperature. Colonies on each plate were counted using OpenCFU software (http://opencfu.sourceforge.net) and cell survival after treatment was expressed as a percentage of the number of colonies in treated plates relative to control plates.

### Tumor xenograft studies

All animal-related procedures and protocols were performed under the Institutional Animal Care and Use Committee (IACUC) at University of South Florida (USF), Wayne State University (WSU), and Shanghai Jiaotong University. All mice were under 24 h/day/7days/week veterinary care; provided food and water ad libitum; and were euthanized at asymptomatic end points. For each mouse model, five or six mice were used. Tumors were implanted to both flanks. So 10-12 tumors were examined at the endpoint. The experiments were not blindly conducted. For Fig. 7c–f: 5 × 10^6^ of control SKOV3 and ES-2 cells or USP10 knockdown SKOV3 and ES-2, in 100 μl of serum-free DMEM or RPMI-1640 medium and Matrigel 1:1 mixture were inoculated subcutaneously (s.c.) to both flanks of six (SKOV3) or five (ES-2) 6–7-week-old female nude mice (one side for control cells, the side for USP10 knockdown cells). Tumor volumes were measured with calipers weekly and calculated as length × width^2^ × 0.5 during the duration of the study. Mice were euthanized and tumors were harvested at the end of the study. The experiments for Fig. 7a, b were performed similarly as Fig. 7c–f, except that female SCID mice were used.

For Fig. 7g–k, in vivo assessment of cisplatin response against USP10 vector control and knockdown for H157 tumor xenografts in SCID mice: 11-week-old female NCR SCID mice (Charles Rivers Labs) were implanted bilaterally s.c. with either H157 parental (vector control) or USP10 KD tumor fragments on day zero. One day post implant, mice were unselectively randomized into various treatment and control groups prior to the initiation of cisplatin treatment. Mice were weighed and assessed daily, and tumors were measured *via* caliper every 3–4 days for the duration of study. One day post last treatment (day 15), all mice were euthanized and tumor tissues were harvested.

Chemotherapy: Mice were administered intravenously (*i.v*.) either vehicle (saline) or cisplatin (diluted from 50 mg/ml pharmaceutical stock; Alvogen, Pine Brook, NJ): 3 mg/kg on a Q4dx4 schedule (total dose:12 mg/kg) or 2 mg/kg on a Q3dx5 schedule (total dose:10 mg/kg). Treatment started one day post implant for all SCID mouse studies (WSU).

Data analysis: tumor volumes were calculated using the formula: volume (mm^3^) = length × width^2^/2. Bilateral implants were added together for total tumor burden per mouse for the determination of %T/C (treatment to control) values. Qualitative measurement of efficacy (%T/C value) = median tumor (treated group)/ median tumor (control group) × 100. This value is calculated on each day of measurement and is an indication of tumor sensitivity to chemotherapy. The values listed in Table S2 correspond to the measurements taken one day post last treatment (day 15).

For murine assessment of USP10 inhibition and cisplatin treatment, 5 × 10^5^ H1299 cells in 100 μl of serum-free DMEM medium and Matrigel 1:1 mixture was inoculated subcutaneously into both flanks of 7-week-old female nude mice. Cisplatin was injected i.p. at 3 mg/kg on a Q5dx4 schedule (every 5 days four times), starting on day 3 for a total dose of 12 mg/kg. USP10 inhibitor P22077 was dissolved in 10% DMSO + 30% PEG 300 + 2% Tween 80 + 58% PBS and injected *i.p.* daily at 15 mg/kg x 37 days. Tumor volume and mouse body weight was measured every 3–4 days. Tumor volumes were measured with calipers and calculated as length × width^2^ × 0.5.

### Tissue microarray

High-density tissue microarrays (TMAs) of ovarian cancer and lung cancer patient samples were purchased from US Biomax, catalog numbers: OV2001 and BC041115. Immunohistochemical (IHC) staining of USP10 and HDAC6 was carried out by Noel Clark at H. Lee Moffitt Cancer Center Pathology Core Facility (Fig. S6). A pathologist scored the immunostaining in a blinded manner. An immunoscore was obtained by calculating the intensity of staining on a scale of 1 to 4 (4 = strong, 3 = moderate, 2 = weak, and 1 = negative) and the percentage of stained ovarian cancer cells, mesothelial cells or lung cancer cells was also measured on a scale of 1 to 4 (1 = 1–20%, 2 = 21–50%, 3 = 51 to 75% and 4 = 76–100%). The representative images for immunoscores 1–4 were shown in Fig. S7. The immunoscore will be obtained using the formula [percentage of immunoreactive cells (1, 2, 3, 4)] × [staining intensity (1, 2, 3, 4)]. Anti-HDAC6 and anti-USP10 IHC staining was evaluated by a pathologist (SVN).

### Immunohistochemical staining

Slides were stained using a Ventana Discovery XT automated system (Ventana Medical System, Tucson, AZ) as per manufacture’s protocol with proprietary reagents. Briefly, slides were deparaffinized on the automated system with EZ Prep solution (Ventana). Heat-induced antigen retrieval method was used in Cell Conditioning 1 (Ventana). The rabbit primary antibody that reacts to USP10 (#ab72486, Abcam, Cambridge, MA) was used at a 1:400 concentration in Dako antibody diluent (Carpenteria, CA) and incubated for 60 min. The Ventana OmniMap anti-rabbit secondary antibody was used for 8 min. The detection system used was the Ventana ChromoMap Kit and slides were then counterstained with Hematoxylin. Slides were then dehydrated and coverslipped as per normal laboratory protocol. Normal kidney was used as control tissue. For anti-HDAC6 staining, heat-induced antigen retrieval method was used in RiboCC (Ventana). The rabbit primary antibody that reacts to HDAC6, (#C0226-1, Assay Biotech, Sunnyvale, CA) was used at a 1:100 concentration in Dako antibody diluent (Carpenteria, CA) and incubated for 32 min. The Ventana UltraMap anti-rabbit Alk phos secondary antibody was used for 12 min. The detection system used was the Ventana ChromoMap Red kit and slides were then counterstained with Hematoxylin. Slides were then dehydrated and coverslipped as per normal laboratory protocol.

### Patient samples

All human subject studies were under the approval of Wayne State University (WSU) Institutional Review Boards (IRBs). RNA was extracted from formalin-fixed paraffin-embedded (FFPE) NSCLC patient sample sections using the High Pure RNA Paraffin Kit from Roche Laboratories (No. 03270289001). The qRT-PCR gene analysis for USP10 was performed on 96 eligible patients that were enrolled in the MADeIT 15005 study^40^ using TaqMan assays (Assay ID for USP10, Hs00382490_m1, Thermo Fisher Scientific). The RPLPO reference gene was used to correct expression values of USP10.

### Statistical analysis

All mice study data were presented as mean ± S.D except that they were stated. Two-sample *t*-tests were performed. For all analyses, *p* values < 0.05 were considered statistically significant.

The clinical data were from a randomized Phase III trial in advanced NSCLC patients^40^. The clinical outcome overall survival (OS) was defined as time from randomization to death due to any cause. Descriptive statistics were provided for the baseline characteristics of patients. A threshold for USP10 RT-PCR expression was searched using a log-rank test within patients treated with platinum-doublet chemotherapy. This threshold was then used to define the USP10 high/low status of patients treated with non-platinum-doublet chemotherapy. A KM plot for subgroups stratified on treatment and marker status was generated. The predictive role of USP10 was evaluated with a Cox model of the treatment and marker interaction for OS adjusted for age, sex, histology, stage, and smoking status. A *p* value of less than 0.05 was considered significant. The analysis was performed with statistical software R, version 3.5.

Survival analysis on TCGA data from cBioPortal was generated as follows. The RNA-seq V2 data for mRNA measurement of USP10 was retrieved for lung adenocarcinoma (*n* = 584) and squamous cell carcinoma (*n* = 501) from TCGA Provisional, where the measurements were preprocessed with RSEM and normalized to *z*-scores. We dichotomized these continuous data into high/low expression with tertiles, where the top one third of measurements were considered high expression. The *TP53* mutation data was retrieved from TCGA *Nat Genet* 2016 (*n* = 1144). The clinical data was from TCGA Provisional, which consists of 584 adenocarcinoma and 511 squamous patients. Three data sets were then merged with patient ID, and a total sample size of 964 patients with complete data was used for survival analysis. The KM plot and log-rank test of OS was performed in subgroups based on *TP53* mutation status. The association between USP10 status (high vs low) and tumor stage (advanced vs early stage) were tested with Chi-square test.

## Supplementary information

supplemental information

Fig S7

supplemental tables

Fig S1.

Fig S2

Fig S3

Fig 4

Fig S5

Fig S6
