# Supplementary material for: The USP10-HDAC6 axis confers cisplatin resistance in non-small cell lung cancer lacking wild-type p53
Source: Cell Death Dis. 2020 May 7;11(5):328. doi: 10.1038/s41419-020-2519-8 (PMC7206099; doi:10.1038/s41419-020-2519-8)
Supplement: Supplementary file 1 — supplemental information [file 41419_2020_2519_MOESM1_ESM.docx]

**Supplemental information**

**The USP10-HDAC6 axis confers cisplatin resistance in non-small cell lung cancer lacking wild-type p53**

Chen Hu, Mu Zhang, Niko Moses, Cong-li Hu, Lisa Polin, Wei Chen, Hyejeong Jang, Joshua Heyza, Joseph A. Caruso, Shengyan Xiang, Steve Patrick, Paul Stemmer, Zhenkun Lou, Wenlong Bai, Chuangui Wang, Gerold Bepler, Xiaohong Mary Zhang

**Supplementary Methods**

**Establishment of the 293T HDAC6 knockout cell line.** The 293T HDAC6 knockout cell line employed for Figure S2 was created using CRISPR/Cas9 (Clustered regularly interspaced short palindromic repeats) method. Briefly, the guide RNA targeting HDAC6 exon 5 (5’-GAAAGGACACGCAGCGATCT-3’) was selected and constructed into a LentiCRISPRv2 vector (Addgene plasmid 52961). The HDAC6-KO vector can also express the codon-optimized Cas9 protein as well as a puromycin resistance gene. The 293T cells transduced with the HDAC6-KO lentiviruses were selected for stable clones using puromycin at 1 µg/ml. The HDAC6-Knockout clones were screened via anti-HDAC6 Western blot analysis.

**Supplementary Statistical Analysis**

**Statistical Method for Figure S4.** For the tumor growth curve, a log-transformed linear mixed effect model was fitted. Wald test was used to assess the growth rate difference between the treatment groups. Z test was used to assess the tumor growth delay at 500mg (500mg was chosen *post hoc*. Not all the treatment groups reach the 1,000mg or 1,500mg). P value less than 0.05 is considered significant. All p values are original without multiple comparisons adjustment. R version 3.4.3 was used.

**Results for Figure S4:**

Tumor growth rate:

H1299-control (Vehicle) 27% per day 95%CI (21%, 32%)

H1299-control (Cisplatin) 18% per day 95%CI (14%, 22%)

H1299-USP10KD (Vehicle) 14% per day 95%CI (11%, 18%)

H1299-USP10KD (Ciplatin) 12% per day 95%CI (9%, 16%)

Pairwise comparison             p.value

H1299-control (Vehicle) vs H1299-control(Cisplatin) 0.0132

H1299-control (Vehicle) vs H1299-USP10KD (Vehicle)    0.0002

H1299-control (Vehicle) vs H1299-USP10KD (Cisplatin)  <.0001

H1299-control(Cisplatin)vs H1299-USP10KD (Vehicle)   0.1307

H1299-control(Cisplatin)vs H1299-USP10KD (Cisplatin) 0.0270

H1299-USP10KD (Vehicle) vs H1299-USP10KD (Cisplatin) 0.4822

Days to 500 mg

H1299-control (Vehicle) 16 days 95%CI (13, 18)

H1299-control(Cisplatin) 21 days 95%CI (18, 24)

H1299-USP10KD (Vehicle)  27 days 95%CI (23, 30)

H1299-USP10KD (Cisplatin) 37 days 95%CI (31, 42)

Pairwise comparison             p.value

H1299-control (Vehicle) vs H1299-control(Cisplatin) 0.0038

H1299-control (Vehicle) vs H1299-USP10KD (Vehicle)  <.0001

H1299-control (Vehicle) vs H1299-USP10KD (Cisplatin) <.0001

H1299-control(Cisplatin)vs H1299-USP10KD (Vehicle)  0.0209

H1299-control(Cisplatin)vs H1299-USP10KD (Cisplatin) <.0001

H1299-USP10KD (Vehicle) vs H1299-USP10KD (Cisplatin) 0.0019

**Conclusions for Figure S4.** USP10KD enhances the efficacy of cisplatin treatment for cell line NCI-H1299 by reducing the tumor growth rate from 18% (14%, 22%) to 12% (9%, 16%) (p value 0.027). Days to tumor volume 500 mg delayed from 21 (18, 24) to 37 (31, 42) (p value <0.0001).

**Supplementary Figure Legends**

**Figure S1. Identification of the USP10 interactome in H1299 cells.** Whole-cell lysates from the Flag-HA-USP10 expressing H1299 cells or vector transfected control H1299 cells were subjected to immunoprecipitation with anti-Flag-M2 agarose beads. The beads were washed and eluted with Flag peptides. Then, the eluted proteins were diluted with lysis buffer, subsequently immunoprecipitated with anti-HA-conjugated agarose beads, and eluted with HA peptides. The elution was subjected to SDS-PAGE for silver staining and the bands, which appeared in the Flag-HA-USP10 lane but not in the control lane, were excised for chromatography-tandem mass spectrometry (LC-MS/MS).

**Figure S2. Overexpression of HDAC6 wild-type or HDAC6 3KR mutant confers cisplatin resistance.** The 293T HDAC6 knockout cells were established as described in the Methods. **A)** The cells were transfected with either empty vector or Flag-tagged wild-type HDAC6 for 24 hours, then treated with 50 μM cisplatin for 0, 24, or 48 hours as indicated. Anti-PARP-1, anti-Flag, and anti-β-actin Western blotting analyses were performed. **B)** The cells were transfected with Flag tagged HDAC6 wild-type or HDAC6 3KR mutant for 24 hours, then treated with 50 μM cisplatin for 0, 36, or 48 hours as indicated. Anti-PARP-1 and anti-β-actin Western blotting analyses were performed.

**Figure S3. H157-USP10 knockdown (KD) xenografts are sensitive to cisplatin, while H157-control xenografts are insensitive to cisplatin.** H157-control and H157-USP10KD were inoculated into SCID mice. Cisplatin or vehicle was injected into the SCID mice, previously implanted with either H157 control or H157 USP10KD tumors *i.v.,* one day after implantation and every 3 days for a total 5 times at the dose of 2 mg/kg, or every 5 days for a total 4 times at the dose of 3 mg/kg. Mice were euthanized 15 days post-implantation. Photos of the tumors were shown in **A)** Tumor volume related to vehicle-treated ones was measured as indicated time points as shown in **B)** (H157-control) and **C)** (H157-USP10KD).

**Figure S4. Knockdown of USP10 in A549 does not affect xenografts growth.** The A549-control or A549-USP10KD cells (1x10^6^ cells per mouse, n=5 each group) were inoculated into the SCID mice subcutaneously and tumor volumes were measured as described in the Methods. Student *t* tests were performed.

**Figure S5. Knockdown of USP10 in H1299 inhibits tumor growth and sensitizes xenografts to cisplatin.** Cisplatin or vehicle was used to treat xenografts (H1299-control or H1299-USP10KD) every 5 days for 4 doses total, at the dose of 3 mg/kg. The treatment started at day 3 after tumor was implanted. Tumor volume was measured as described in the Methods.

**Figure S6. The layouts of ovarian cancer and lung cancer TMAs. A)** The layout of the lung cancer TMA with 120 cases/cores (left panel) and the IHC staining for anti-USP10 (middle panel) and anti-HDAC6 (right panel). **B)** The layout of the ovarian cancer TMA with 200 cases/cores (left panel) and the IHC staining for anti-USP10 (middle panel) and anti-HDAC6 (right panel). Both TMAs were obtained from US Biomax. Clinical information for each section, including patient age, sex, pathology diagnosis, grade, stage and type, can be found online ([https://www.biomax.us/TissueArrays](https://www.biomax.us/Tissue_Arrays)) through the TMA names.

**Figure S7. The representative IHC intensities.** The representative anti-USP10 and anti-HDAC6 IHC staining images from ovarian cancer TMA for intensities of negative (1), weak (2), moderate (3), and strong (4) are shown.
