# Supplementary figures and images for: The USP10-HDAC6 axis confers cisplatin resistance in non-small cell lung cancer lacking wild-type p53
Source: Cell Death Dis. 2020 May 7;11(5):328. doi: 10.1038/s41419-020-2519-8 (PMC7206099; doi:10.1038/s41419-020-2519-8)

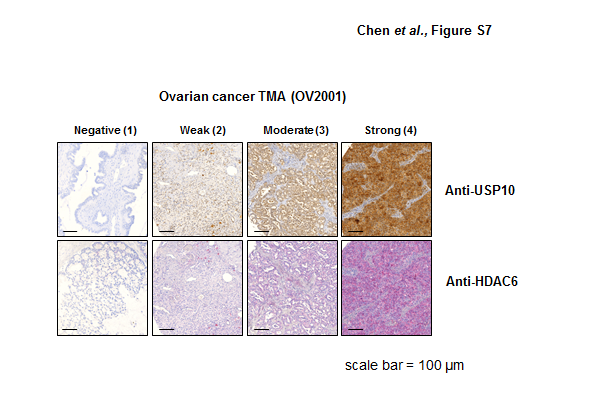

Supplement: Supplementary file 2 — Fig S7 [file 41419_2020_2519_MOESM2_ESM.tif]

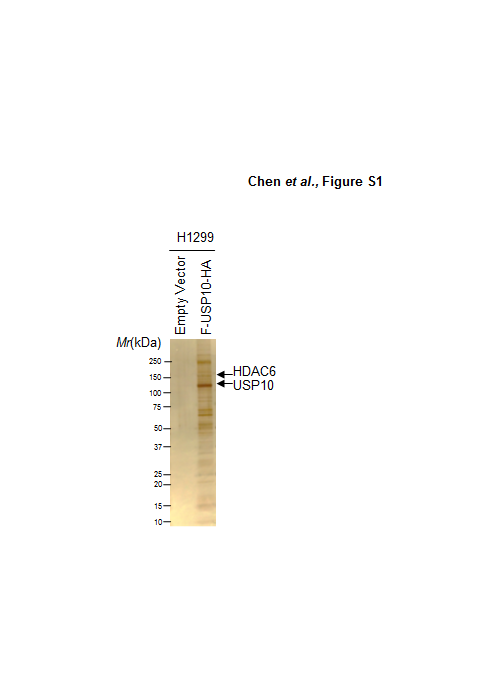

Supplement: Supplementary file 4 — Fig S1. [file 41419_2020_2519_MOESM4_ESM.tif]

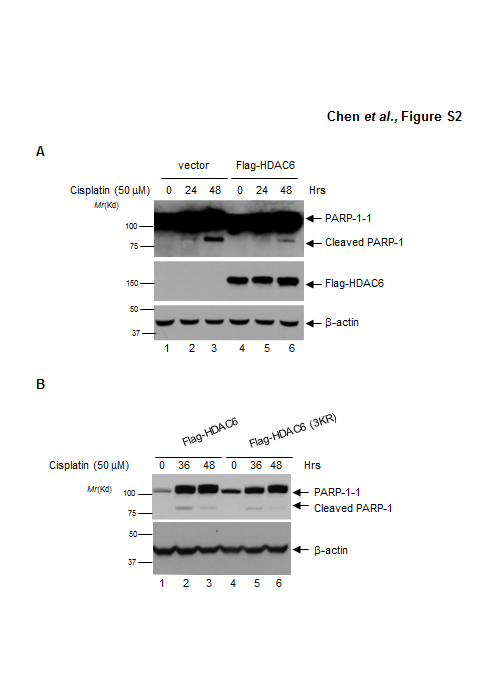

Supplement: Supplementary file 5 — Fig S2 [file 41419_2020_2519_MOESM5_ESM.tif]

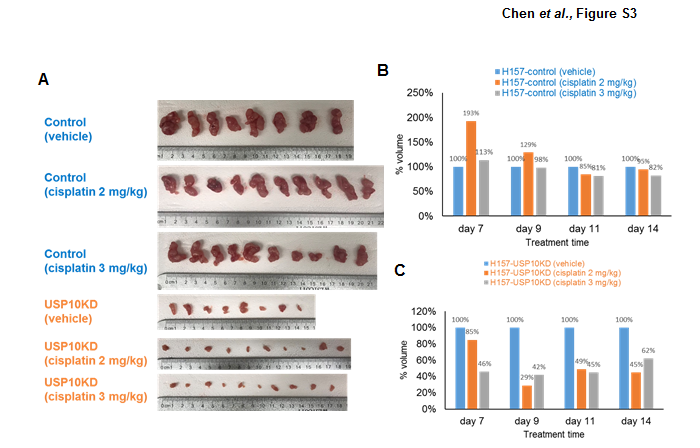

Supplement: Supplementary file 6 — Fig S3 [file 41419_2020_2519_MOESM6_ESM.tif]

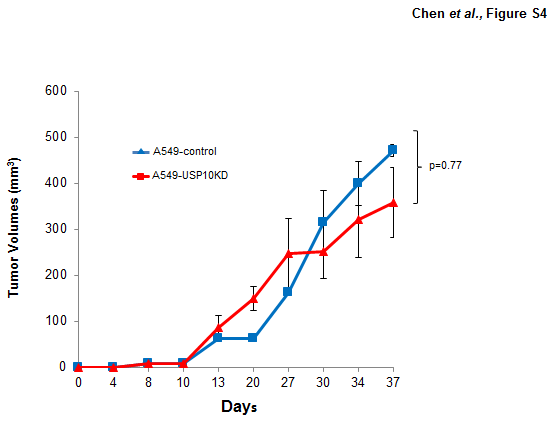

Supplement: Supplementary file 7 — Fig 4 [file 41419_2020_2519_MOESM7_ESM.tif]

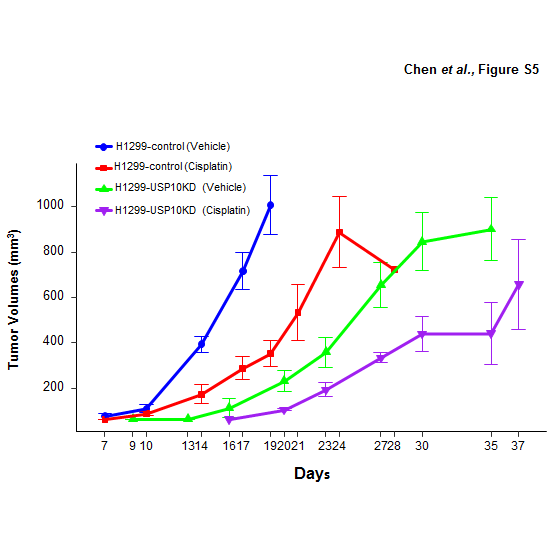

Supplement: Supplementary file 8 — Fig S5 [file 41419_2020_2519_MOESM8_ESM.tif]

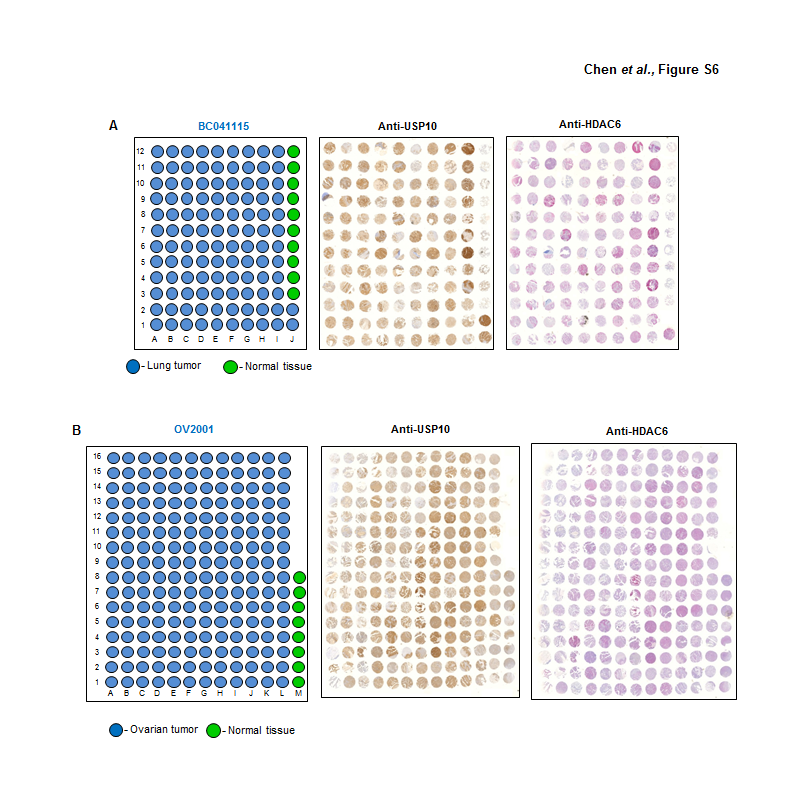

Supplement: Supplementary file 9 — Fig S6 [file 41419_2020_2519_MOESM9_ESM.tif]
