# Supplementary material for: The USP10-HDAC6 axis confers cisplatin resistance in non-small cell lung cancer lacking wild-type p53
Source: Cell Death Dis. 2020 May 7;11(5):328. doi: 10.1038/s41419-020-2519-8 (PMC7206099; doi:10.1038/s41419-020-2519-8)
Supplement: Supplementary file 3 — supplemental tables [file 41419_2020_2519_MOESM3_ESM.docx]

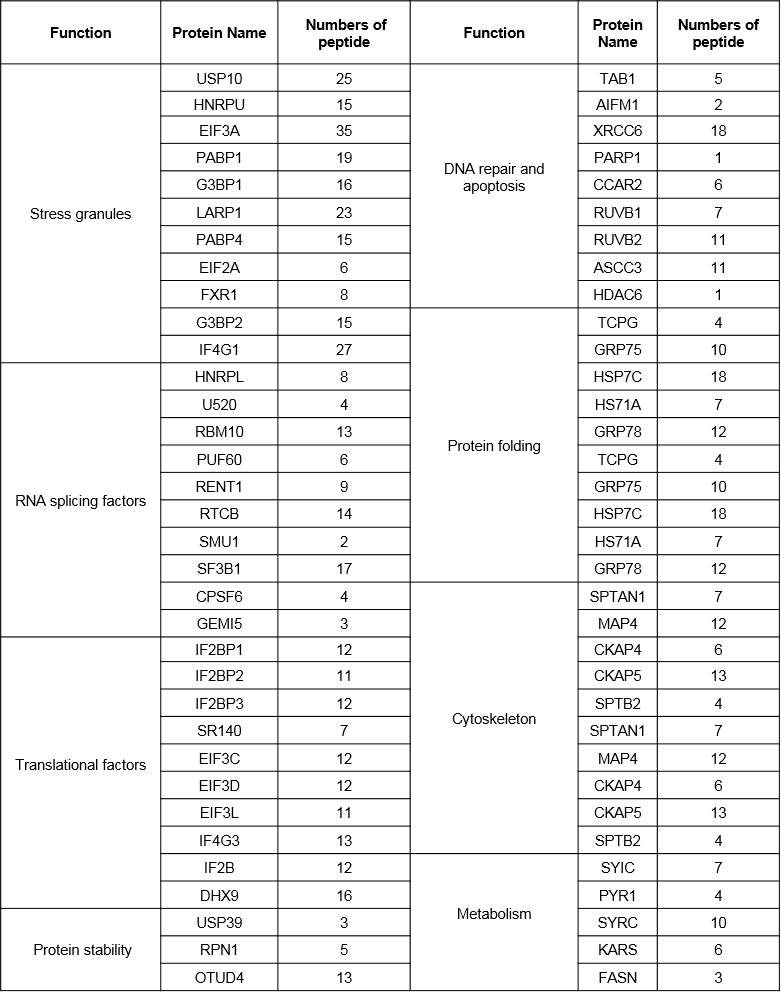


**Table S1. The USP10 interactome in H1299 cells**


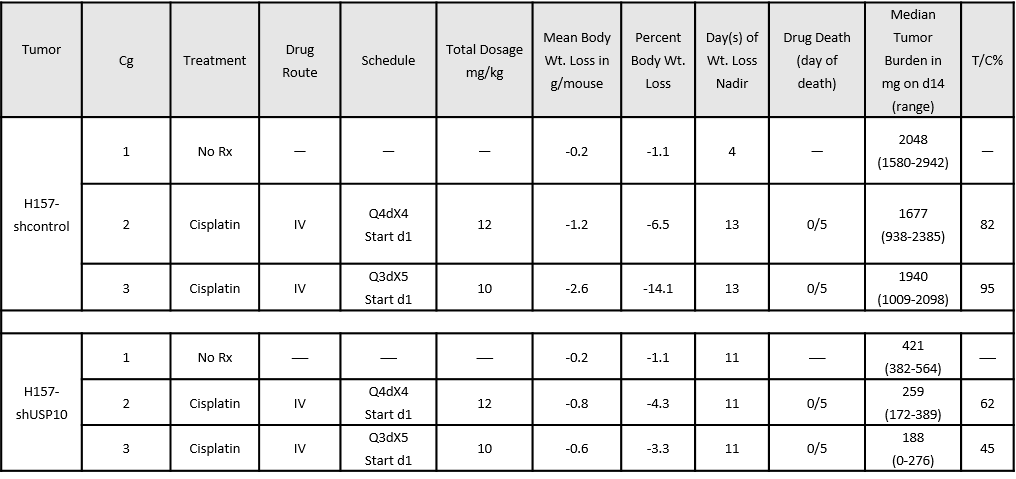


**Table S2. The scheme of H157 xenograft experiments for Figure 7g-i**


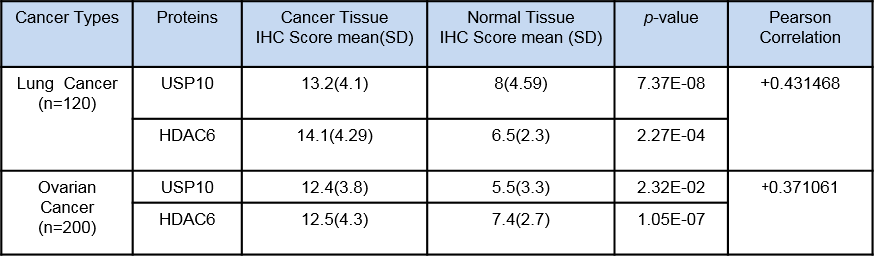


**Table S3. USP10 and HDAC6 co-expression in lung and ovarian patient samples**

F, female; M, male; W, white; B, black; Unk, unkown.


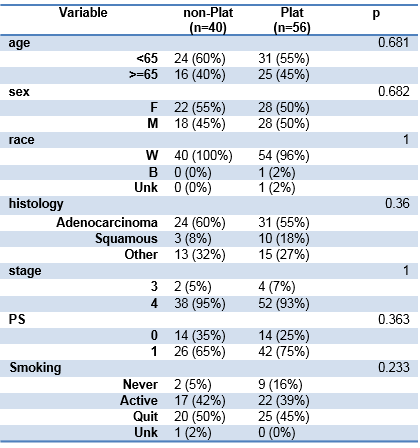


**Table S4. Patient characteristics by treatment groups**


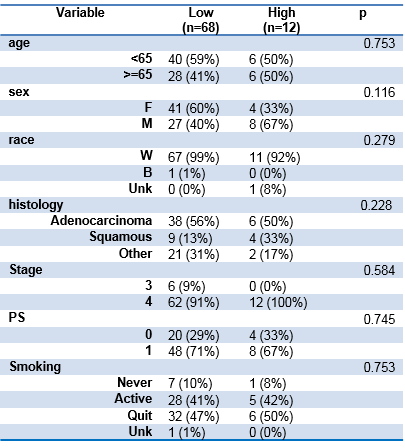


**Table S5. Patient characteristics by USP10 status**

F, female; M, male; W, white; B, black; Unk, unkown.
